# Genetic architecture of end-use quality traits in soft white winter wheat

**DOI:** 10.1186/s12864-022-08676-5

**Published:** 2022-06-14

**Authors:** Meriem Aoun, Arron H. Carter, Craig F. Morris, Alecia M. Kiszonas

**Affiliations:** 1 Department of Crop and Soil Sciences, Washington State University, Pullman, WA 99164 USA; 2 Currently Department of Entomology and Plant Pathology, Oklahoma State University, Stillwater, OK 74078 USA; 3 USDA-ARS Western Wheat & Pulse Quality Laboratory, Washington State University, E-202 Food Quality Building, Pullman, WA 99164 USA

**Keywords:** Soft white winter wheat, End-use quality, Molecular markers, Association mapping

## Abstract

**Background:**

Genetic improvement of end-use quality is an important objective in wheat breeding programs to meet the requirements of grain markets, millers, and bakers. However, end-use quality phenotyping is expensive and laborious thus, testing is often delayed until advanced generations. To better understand the underlying genetic architecture of end-use quality traits, we investigated the phenotypic and genotypic structure of 14 end-use quality traits in 672 advanced soft white winter wheat breeding lines and cultivars adapted to the Pacific Northwest region of the United States.

**Results:**

This collection of germplasm had continuous distributions for the 14 end-use quality traits with industrially significant differences for all traits. The breeding lines and cultivars were genotyped using genotyping-by-sequencing and 40,518 SNP markers were used for association mapping (GWAS). The GWAS identified 178 marker-trait associations (MTAs) distributed across all wheat chromosomes. A total of 40 MTAs were positioned within genomic regions of previously discovered end-use quality genes/QTL. Among the identified MTAs, 12 markers had large effects and thus could be considered in the larger scheme of selecting and fixing favorable alleles in breeding for end-use quality in soft white wheat germplasm. We also identified 15 loci (two of them with large effects) that can be used for simultaneous breeding of more than a single end-use quality trait. The results highlight the complex nature of the genetic architecture of end-use quality, and the challenges of simultaneously selecting favorable genotypes for a large number of traits. This study also illustrates that some end-use quality traits were mainly controlled by a larger number of small-effect loci and may be more amenable to alternate selection strategies such as genomic selection.

**Conclusions:**

In conclusion, a breeder may be faced with the dilemma of balancing genotypic selection in early generation(s) versus costly phenotyping later on.

**Supplementary Information:**

The online version contains supplementary material available at 10.1186/s12864-022-08676-5.

## Introduction

End-use quality improvement in soft white wheat (*Triticum aestivum* L.) is one of the primary objectives of wheat breeding programs. End-use quality is complex and involves multiple traits. The key end-use quality parameters in soft white wheat include softer kernels, lower grain protein content and gluten strength, less damaged starch, lower non-starch polysaccharides that lead to decreased water absorption capacity, larger cookies diameter and cake volume. For some soft wheat products, starch paste viscosity is a key quality trait.

In breeding programs, end-use quality phenotyping is laborious, expensive, time consuming and requires a large amount of grain. Consequently, selection for end-use quality is often delayed until later breeding stages [1, 2]. Since most end-use quality traits are predominantly controlled by genetic factors [3–5], a better understanding of the underlying genetic architecture of the various traits can support strategies for both phenotypic and genotypic selection, including an assessment of the potential effectiveness of marker-assisted selection. Analysis of marker trait associations have identified numerous quantitative trait loci (QTL) for different end-use quality traits distributed across all 21 wheat chromosomes [2, 4, 6–23]. However, most of these studies were performed in hard wheat (bread wheat) and these investigations [2, 4, 17, 22, 23] were performed in soft wheat. Soft white wheat has unique milling and baking parameters which are aimed at making food products such as cookies and cakes [24, 25].

A number of end-use quality traits are influenced by the effect(s) of major genes. For example, the genetic architecture of grain hardness is primarily controlled by the puroindolines, of gluten strength by the high molecular weight glutenins, and of starch paste viscosity by the granule bound starch synthase (‘waxy’) genes [1, 26]. However, these major genes are often fixed in elite breeding populations due to parent selection, or early generation phenotypic and/or genotypic selection and do not sufficiently account for the levels of end-use quality required for cultivar release nor for the range of variation observed among breeding populations [25].

A number of mapping studies for end-use quality were performed in bi-parental populations and in some cases one or both parents were either poorly adapted or would not constitute ‘elite’ germplasm for applied plant breeding [9, 11, 13]. Additionally, the bi-parental genetic structure limits QTL mapping resolution. Genome-wide association mapping (GWAS) can overcome these limitations by using historical recombination events that occur throughout the germplasm evolution and using elite breeding germplasm from the breeding program of interest.. In this study we implemented GWAS using recent breeding lines and cultivars from the Washington State University (WSU) soft white winter wheat breeding program to investigate the underlying genetic architecture of phenotypic variation of 14 end-use quality traits in 672 soft white winter wheat genotypes. We identified end-use quality associated single nucleotide polymorphism (SNP) markers using GWAS and identified large effect QTL. These QTL contribute to better understanding of the underlying genetic architecture of end-use quality in soft white wheat and provide an objective assessment as to the potential for marker assisted selection (MAS) versus other genotypic and phenotypic selection strategies.

## Materials and methods

### Plant materials

A total of 672 soft white winter wheat breeding lines and cultivars were used in this study. The breeding lines were F_4:5_ lines and double haploid lines selected from different crosses to represent the diversity present in the WSU winter wheat breeding program. The genotypes and the environments in which the lines were grown were described in Aoun et al. [27]. In brief, this germplasm was evaluated in 29 environments (year-location combinations). Genotypes were grown from 2015 to 2019 in seven locations in Washington State (WA), USA including Pullman, Lind, Davenport, Ritzville, Waterville, Walla Walla, and Dayton. In this dataset, there were 1–7 nurseries per environment with a total of 76 nurseries. From each nursery, a single sample from one replicate per genotype was evaluated for end-use quality traits. The dataset was unbalanced with some shared lines between environments. The connectivity between environments in terms of genotypes was described in Aoun et al. [27]. There were 43 genotypes (out of the 672) evaluated for end-use quality in more than one-quarter of the environments [27].

### Phenotypic data

The wheat genotypes were evaluated for 14 end-use quality traits that are classified into four categories which are grain characteristics, milling traits, flour characteristics, and baking parameters. The phenotypic and genotypic data were retrieved from Aoun et al. [27] which investigated genotype × environment interactions and tested the performance of genomic prediction for the 14 end‐use quality traits. Traits associated with grain characteristics included Single Kernel Characterization System (SKCS) hardness, SKCS size, SKCS weight, test weight, and grain protein content. SKCS hardness is a key determinant of end-use quality where hard wheat is mainly used for making bread, and soft wheat is primarily used for making cookies, cakes, and confectionery products [1, 28, 29]. In the grain market, test weight and grain protein content are the two main parameters. High test weight, which is correlated with kernel weight and size [30, 31], usually leads to higher milling performance [32].

Milling traits included break flour yield, flour yield, flour ash content, and milling score. Break flour yield was calculated as the percent of flour recovered from the break rolls, whereas flour yield (‘straight grade’) was determined as the proportion of grain recovered as flour (break plus reduction flour). Flour ash content is the minerals remaining after flour combustion. Milling score was a function of both flour yield and flour ash content [33]. Higher break flour yield, flour yield, and milling score are desirable in soft wheat. Higher inclusion of bran reduces the functionality of most doughs and batters [34]. As such, mineral content of flours (ash) serves as a proxy for bran contamination and lower flour ash is preferred.

Flour functionality plays an important role in baking performance. Flour parameters included flour protein content, flour sodium dodecyl sulfate sedimentation volume (SDS sedimentation), water solvent retention capacity (water SRC), and flour swelling volume (FSV). Unlike bread, soft white wheat products require lower grain/flour protein content, weaker gluten strength (lower SDS sedimentation volume), and low water SRC. FSV is an end-use quality parameter associated with the amount of amylose and amylopectin components in endosperm starch [35] and needs to be high for making some Asian-style noodles [1, 36]. The baking parameter cookie diameter is considered an important indicator of the overall quality of soft wheat [28, 37] and has been a key selection trait in soft wheat breeding programs.

These end-use quality traits were measured following the procedures from the American Association of Cereal Chemists International [38] and as described by Aoun et al. [27]. The data set was analyzed using mixed linear model in the R package lme4 [39, 40]. The environments were considered random, while genotypes were fitted as fixed in the model. For each trait, best linear unbiased estimators (BLUEs) of the genotypes were extracted from the mixed linear model and used for further statistical analysis. Broad sense heritability (*H*^*2*^_Cullis_) and correlations between traits were previously described by Aoun et al. [27].

### Genotyping

Genotyping-by-sequencing (GBS) [41] was used to genotype the 672 soft white wheat breeding lines and cultivars. The genotypic data for the 672 genotypes were previously provided by Aoun et al. [27]. The GBS was performed at the North Carolina State University Genomic Sciences Laboratory in Raleigh, NC, USA. The sequence reads were aligned to the *T. aestivum* RefSeq v1.0 reference genome [42] and SNP data were filtered for minor allele frequency (MAF) ≥ 5%, missing data ≤ 30%, and heterozygous frequency ≤ 15%. From this, 40,518 SNPs were used for further analysis. Missing datapoints in the SNP data were imputed using the expectation–maximization algorithm implemented in the package rrBLUP [43] in R version 4.0.2 [44].

### Population structure and linkage disequilibrium

To visualize the population structure in the 672 genotypes, principal component analysis (PCA) was performed using the ‘prcomp’ function in R based on 40,518 SNPs. The population structure was visualized using the first two principal components (PCs) that explained the highest percentage of variation. Pairwise linkage disequilibrium (LD) between SNPs (*r*^2^) was estimated using TASSEL v5 [45] by applying a sliding window of 50 markers. The *r*^2^ values of marker pairs were plotted against the physical distances in Mega base pairs (Mb) after randomly selecting 10% of the total SNP pairs. To visualize the LD decay across the genome and for each of the 21 chromosomes, a locally estimated scatterplot smoothing (LOESS) curve was fitted using the function ‘geom_smooth’ in R package ggplot2 [46]. The *r*^*2*^ threshold was derived from the 95^th^ percentile of the distribution of unlinked *r*^2^ (for markers on different chromosomes) [47] that were significant at the 99.99% level of confidence. The *r*^*2*^ threshold is the value beyond which LD was likely to be caused by genetic linkage. The intersection of the horizontal line at the *r*^*2*^ threshold value with the LOESS curve on the LD scatter plot was considered as the estimate of the extent of LD across the genome (genome-wise LD decay plot) and across each chromosome (chromosome-wise LD decay plot).

### Genome-wide association mapping

The BLUEs for each trait were considered as the phenotype in the GWAS. Association mapping was performed using three models 1) mixed linear model (MLM), 2) Fixed and random model Circulating Probability Unification (FarmCPU) [48], and 3) Bayesian-information and Linkage-disequilibrium Iteratively Nested Keyway (BLINK) [49] implemented in the GAPIT R package [50]. The single-locus MLM is the most widely used in association mapping studies. However, it tests one marker at a time and therefore is likely to increase the number of false negatives for complex traits [22, 51]. Multi-locus models such as FarmCPU were proposed to overcome this problem. FarmCPU iteratively uses fixed and random models in which the identified significant SNPs from the iterations are fitted as cofactors [48]. FarmCPU was reported to control for false negatives and false positives without causing model overfitting. BLINK was derived from the FarmCPU method with a few modifications. BLINK does not assume that causal genes are evenly distributed across the genome. It also works directly on markers instead of bins and excludes markers in LD with the most significant markers. BLINK uses Bayesian Information Content (BIC) of a fixed effect model to approximate the maximum likelihood of a random effect model to select marker trait associations (MTAs).

The GWAS models considered family relatedness (Kinship matrix or K matrix) [52] and population structure (Q matrix). K matrix was included in all GWAS models, whereas the optimal number of principal components (PCs) in the Q matrix were determined based on quantile–quantile (Q-Q) plots that visualize the expected -log_10_ (*P*) versus the observed -log_10_ (*P*). The number of PCs included in the GWAS models was limited to the first four PCs. Manhattan plots for MTAs were visualized using the R package ‘qqman’ [53]. MTAs were considered significant at a false discovery rate (FDR) [54] of ≤ 0.05.

Based on the *T. aestivum* RefSeq v1.0 reference genome assembly (https://wheat.pw.usda.gov/jb/?data=/ggds/whe-iwgsc2018), we identified annotated genes within the genomic regions of significant SNPs that exhibit large effects (significantly impacted trait values based on Tukey’s HSD test, had large impact on the phenotype, and were unlikely to be false positives, i.e., MAF ≥ 7%). In this search, we considered only high-confidence annotated genes located within a few kilobase pairs before and after the associated SNP physical positions. The putative biological functions of the candidate genes were retrieved from this website https://wheat-urgi.versailles.inra.fr/Seq-Repository/Annotations. In addition, we extracted MTAs available at the International Wheat Genome Sequencing Consortium (IWGSC) sequence repository that were within the genomic regions of large effect MTAs. Furthermore, markers (SNP, simple sequence repeat: SSR, Diversity Arrays Technology: DArT) associated with end-use quality in previous genetic studies [2, 4, 6–17, 19–22] and physically close (based on *T. aestivum RefSeq* v1.0 reference genome) to all MTAs in this study were determined.

## Results

### Phenotypic data

**Fig. 1:**
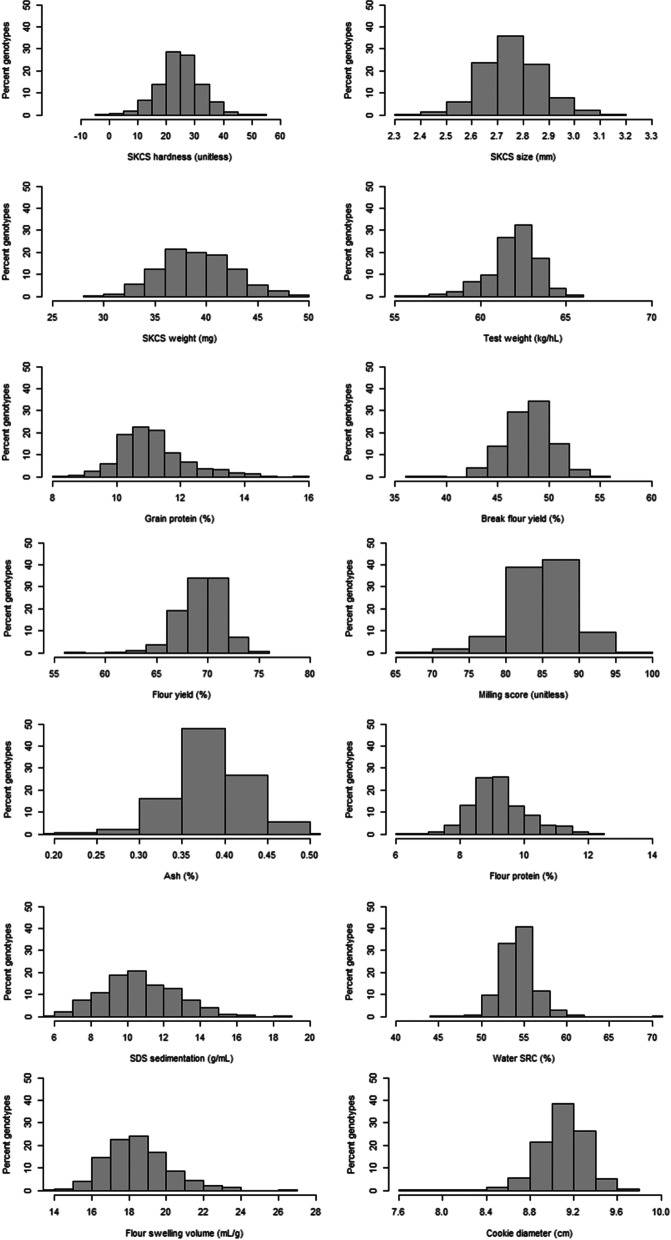
Distributions of best linear unbiased estimators (BLUEs) for 14 end-use quality traits in 672 soft white winter wheat genotypes

The distributions of BLUEs for each of the 14 end-use quality traits are illustrated in Fig. 1. There were continuous phenotypic distributions for all end-use quality traits. For grain characteristics, BLUEs ranged from -0.8 to 54.1 for SKCS hardness, 2.1 to 3.2 mm for SKCS size, 28.5 to 48.3 mg for SKCS weight, 55.6 to 65.4 kg/hL for test weight, and 8.3 to 15.7% for grain protein. For milling traits, BLUEs ranged from 36.5 to 54.9% for break flour yield, 57.1 to 74.3% for flour yield, 0.20 to 0.51% for flour ash, and 67.0 to 98.6 for milling score. For flour parameters, BLUEs ranged from 6.4 to 12.4% for flour protein, 3.4 to 18.2 g/mL for SDS sedimentation, 44.5 to 73.1% for water SRC, and 11.6 to 26.2 mL/g for FSV. For cookie diameter, BLUEs ranged from 7.8 to 9.7 cm. For all of these traits, differences in phenotypes would be considered to be industrially significant, with many values below minimum targets [25]. Moderate to high broad sense heritability (*H*^*2*^ = 0.46–0.70) was observed for all traits except for grain and flour protein content (*H*^*2*^ = 0.18 to 0.19) [27].

### Population structure and linkage disequilibrium

Of the 40,518 SNPs, there were 14,102 (34.8%) SNPs on the A genome, 16,626 (41.0%) SNPs on the B genome, 8,656 (21.4%) SNPs on the D genome, and 1,134 (2.8%) SNPs on unaligned (UN) chromosome(s). PCA based on the first two PCs showed minimal clustering in wheat genotypes, which was expected since the plant materials in this study were from the same wheat breeding program (Supplementary Fig. S1). The first 10 PCs accounted cumulatively for 26.3% of the variation. The first four PCs explained 5.3%, 4.0%, 3.3% and 3.0% of variation, respectively. The genome-wise LD dropped to an *r*^*2*^ threshold of 0.1 within 6.5 Mb on average (Supplementary Fig. S2). LD decayed to 0.1 at ~ 2.5–5.0 Mb for chromosomes on the A genome, to 5.0–10 Mb for chromosomes on the B and the D genome (Supplementary Fig. S3).

### Genome-wide association mapping

#### GWAS model selection

The best models within each method were selected based on examination of Q-Q plots. For MLM, we selected MTAs from the K (Kinship) model. Using FarmCPU, K + 2PCs (Kinship and Q based on the first two PCs) was selected to model SKCS hardness, SKCS weight, grain protein content, flour yield, flour ash, milling score, SDS sedimentation, water SRC, and cookie diameter, whereas K + 3PCs (Kinship and Q based on the first three PCs) was selected to model the remaining traits, SKCS size, test weight, break flour yield, flour protein content, and FSV. For BLINK, we selected K + 4PCs (Kinship and Q based on the first four PCs) for all traits.

In contrast to the Q-Q plots generated from FarmCPU models, the Q-Q plots from MLM and BLINK did not show a sharp deviation of the observed *P*-value distribution from the expected *P*-value distribution (Supplementary Fig. S4, S5, S6). These results suggest that FarmCPU provided a better control of false negatives and false positives compared to MLM and BLINK. Thus, only association mapping results from FarmCPU will be discussed in this study (Tables 1,2,3,4 , Supplementary Fig. S7). MTAs generated from MLM and BLINK are provided in Supplementary Table S1, S2.

**Table 1:** Summary of SNP markers associated with grain characteristics in 672 soft white winter wheat genotypes

Trait^a^	SNP^b^	Chromosome	Position (bp)^c^	Alleles^d^	MAF^e^	*P* value^f^	FDR^g^	Effect
SKCS hardness	*S1A_583894689*	1A	583,894,689	**T**/C	0.28	1.75E-05	4.44E-02	1.04
SKCS hardness	*S1B_635869100*	1B	635,869,100	A/**G**	0.24	3.84E-08	5.19E-04	-1.46
SKCS hardness	*S3A_690786387*	3A	690,786,387	**A**/G	0.23	4.73E-06	1.60E-02	1.72
SKCS hardness	*S3B_718850970*	3B	718,850,970	C/**G**	0.46	7.04E-09	1.43E-04	-1.34
SKCS hardness	*S4D_509798252*	4D	509,798,252	A/**G**	0.38	1.32E-05	3.55E-02	-0.97
SKCS hardness	***S5A_480515221***	5A	480,515,221	C/**T**	0.14	2.91E-19	1.18E-14	-3.34
SKCS hardness	*S5B_549556474*	5B	549,556,474	G/**T**	0.06	1.45E-07	1.18E-03	-2.49
SKCS hardness	*S6B_11537871*	6B	11,537,871	C/**T**	0.05	5.51E-07	3.19E-03	-3.51
SKCS hardness	*S6B_130163859*	6B	130,163,859	A**/G**	0.18	4.98E-07	3.19E-03	-1.47
SKCS hardness	*S6B_233122036*	6B	233,122,036	**T**/C	0.07	7.69E-07	3.90E-03	2.10
SKCS hardness	***S6B_705613777***	6B	705,613,777	**G**/A	0.08	1.36E-06	6.15E-03	2.14
SKCS hardness	*S7A_12011069*	7A	12,011,069	**T**/A	0.05	9.44E-06	2.73E-02	2.85
SKCS hardness	*S7A_514892901*	7A	514,892,901	A/**T**	0.12	1.56E-06	6.32E-03	-1.78
SKCS hardness	*S7B_643501407*	7B	643,501,407	**A**/G	0.46	5.79E-08	5.86E-04	1.37
SKCS hardness	*S7D_56065854*	7D	56,065,854	**C**/G	0.17	9.33E-06	2.73E-02	1.32
SKCS hardness	*S7D_617377539*	7D	617,377,539	**C**/T	0.06	3.91E-06	1.44E-02	2.27
SKCS size	*S1B_569507932*	1B	569,507,932	T/**C**	0.47	1.19E-06	5.36E-03	0.02
SKCS size	*S2B_154846350*	2B	154,846,350	T/**G**	0.40	1.72E-06	6.96E-03	0.02
SKCS size	*S2D_563799166*	2D	563,799,166	**G**/T	0.07	8.65E-06	2.50E-02	-0.04
SKCS size	*S2D_586961640*	2D	58,6961,640	A/**G**	0.20	3.38E-06	1.05E-02	0.03
SKCS size	*S3A_60689591*	3A	60,689,591	**T**/C	0.32	3.84E-07	2.25E-03	-0.02
SKCS size	*S3A_721092685*	3A	721,092,685	C/**G**	0.17	2.26E-07	1.96E-03	0.03
SKCS size	*S4B_578521140*	4B	578,521,140	T/**C**	0.23	2.91E-08	3.93E-04	0.03
SKCS size	*S5A_578074731*	5A	578,074,731	**A**/G	0.21	4.99E-07	2.53E-03	-0.03
SKCS size	*S5A_583031341*	5A	583,031,341	**C**/T	0.42	2.28E-06	7.80E-03	-0.02
SKCS size	*S5A_636959783*	5A	636,959,783	**T**/C	0.17	2.31E-06	7.80E-03	-0.02
SKCS size	*S5B_615969532*	5B	615,969,532	G/**A**	0.08	3.89E-07	2.25E-03	0.04
SKCS size	*S6B_126632693*	6B	126,632,693	T/**G**	0.05	1.31E-08	2.65E-04	0.04
SKCS size	*S6B_336606108*	6B	336,606,108	**T**/G	0.17	2.42E-07	1.96E-03	-0.02
SKCS size	*S6B_583281710*	6B	583,281,710	G/**A**	0.11	2.86E-10	1.16E-05	0.04
SKCS weight	*S1B_427530823*	1B	427,530,823	**T**/C	0.15	5.42E-07	4.39E-03	-0.87
SKCS weight	*S2A_165870782*	2A	165,870,782	A/**T**	0.38	8.34E-07	5.63E-03	0.64
SKCS weight	*S2B_18326207*	2B	18,326,207	C/**T**	0.23	4.55E-07	4.39E-03	0.63
SKCS weight	***S2B_533178165***	2B	533,178,165	**C/**G	0.09	4.42E-07	4.39E-03	-1.11
SKCS weight	*S2D_563799166*	2D	563,799,166	**G**/T	0.07	1.16E-05	5.22E-02	-1.04
SKCS weight	*S2D_613442636*	2D	613,442,636	G/**T**	0.05	1.77E-06	8.96E-03	1.36
SKCS weight	*S6B_583281710*	6B	583,281,710	G/**A**	0.11	1.77E-07	4.39E-03	0.75
SKCS weight	*S7B_686004231*	7B	686,004,231	**G**/A	0.09	3.31E-07	4.39E-03	-1.15
SKCS weight	*S7D_7495567*	7D	7,495,567	G/**T**	0.05	1.14E-06	6.57E-03	1.42
Test weight	*S2A_762292662*	2A	762,292,662	**T/**G	0.23	8.08E-06	2.89E-02	-0.31
Test weight	***S4B_413497949***	4B	413,497,949	**C**/T	0.09	4.02E-08	5.43E-04	-0.39
Test weight	*S5A_611568887*	5A	611,568,887	A/**G**	0.25	1.79E-08	3.62E-04	0.24
Test weight	*S5B_506953332*	5B	506,953,332	**T**/A	0.21	1.76E-06	1.42E-02	-0.21
Test weight	*S5B_550910513*	5B	550,910,513	T/**C**	0.43	3.72E-06	2.15E-02	0.19
Test weight	*S5B_605171604*	5B	605,171,604	T/**C**	0.09	4.76E-06	2.15E-02	0.27
Test weight	*S6A_46675024*	6A	46,675,024	**C**/G	0.06	5.32E-06	2.15E-02	-0.36
Test weight	*S6A_559428977*	6A	559,428,977	G/**A**	0.26	2.45E-09	9.93E-05	0.27
Test weight	*S6B_510608440*	6B	510,608,440	G/**A**	0.08	4.91E-06	2.15E-02	0.40
Test weight	*S7A_669715941*	7A	669,715,941	**T**/C	0.14	3.66E-06	2.15E-02	-0.29
Test weight	*S7B_40194878*	7B	40,194,878	G/**C**	0.18	2.26E-07	2.29E-03	0.25
Test weight	*S7B_636744313*	7B	636,744,313	T/**A**	0.17	8.54E-06	2.89E-02	0.28
Grain protein	*S1A_585175145*	1A	58,517,5145	**G**/C	0.10	1.33E-05	4.48E-02	0.28
Grain protein	***S1B_46883868***	1B	46,883,868	**C/**A	0.09	1.04E-10	4.22E-06	0.38
Grain protein	*S1B_633974958*	1B	633,974,958	A/**G**	0.45	9.35E-08	7.58E-04	-0.18
Grain protein	*S2A_612836091*	2A	612,836,091	C/**T**	0.05	5.91E-06	2.18E-02	-0.31
Grain protein	*S3B_405161312*	3B	405,161,312	T/**C**	0.27	2.74E-06	1.20E-02	-0.19
Grain protein	*S3B_690878020*	3B	690,878,020	T/**C**	0.44	1.72E-06	9.94E-03	-0.14
Grain protein	*S4A_352495200*	4A	352,495,200	G/**A**	0.10	1.61E-06	9.94E-03	-0.23
Grain protein	*S4B_63121316*	4B	63,121,316	**C**/T	0.10	3.36E-08	3.41E-04	0.31
Grain protein	*S5D_543253602*	5D	543,253,602	G/**A**	0.08	1.85E-09	2.50E-05	-0.38
Grain protein	*S6D_471179733*	6D	471,179,733	**A**/C	0.05	2.95E-06	1.20E-02	0.52
Grain protein	*S7A_731026067*	7A	731,026,067	**A**/T	0.26	1.43E-09	2.50E-05	0.27
Grain protein	*SUN_86449317*	UN	86,449,317	**T**/G	0.19	1.45E-05	4.53E-02	0.22
Grain protein	*SUN_351152321*	UN	351,152,321	A/**G**	0.31	2.75E-06	1.20E-02	-0.19

^a^SKCS, Single Kernel Characterization System

^b^Significant single-nucleotide polymorphism (SNP). Markers in bold are markers with large effects

^c^Physical position of SNP sequence based on Wheat Chinese Spring IWGSC RefSeq v1.0

^d^SNP major allele/minor allele, the allele in bold is the favorable allele in soft white wheat (associated with higher phenotypic values for SKCS size, SKCS weight, and test weight and lower phenotypic values for SKCS hardness and grain protein content)

^e^Minor allele frequency of the SNP

^f^*P* value of the significant SNP

^g^False discovery rate of the significant SNP

**Table 2:** Summary of SNP markers associated with milling traits in 672 soft white winter wheat genotypes

Trait	SNP^a^	Chromosome	Position (bp)^b^	Alleles^c^	MAF^d^	*P* value^e^	FDR^f^	Effect
Break flour yield	*S1B_100055026*	1B	100,055,026	T/**C**	0.08	2.96E-07	2.40E-03	1.00
Break flour yield	***S1B_653681752***	1B	65,368,1752	**G/**T	0.14	1.32E-13	3.52E-09	-1.05
Break flour yield	*S2A_678005650*	2A	678,005,650	C/**T**	0.31	1.74E-13	3.52E-09	0.55
Break flour yield	*S3B_630394456*	3B	630,394,456	G/**T**	0.06	6.94E-07	4.69E-03	0.79
Break flour yield	*S4A_584538827*	4A	584,538,827	**A**/G	0.11	7.69E-11	1.04E-06	-0.87
Break flour yield	*S4B_611852943*	4B	611,852,943	G/**C**	0.08	6.37E-09	6.45E-05	0.70
Break flour yield	*S5B_508665777*	5B	508,665,777	**T**/C	0.07	3.67E-06	1.65E-02	-0.62
Break flour yield	*S7A_34527313*	7A	34,527,313	G/**C**	0.33	9.84E-06	3.63E-02	0.27
Break flour yield	*S7A_642899331*	7A	642,899,331	**C**/G	0.14	1.23E-06	7.13E-03	-0.44
Break flour yield	*S7A_732594008*	7A	732,594,008	**A**/G	0.23	1.60E-06	8.11E-03	-0.41
Break flour yield	*S7D_56065854*	7D	56,065,854	**C**/G	0.17	4.55E-06	1.84E-02	-0.44
Flour yield	*S1A_9128313*	1A	9,128,313	G/**T**	0.39	1.09E-06	4.43E-03	0.38
Flour yield	*S1A_15686346*	1A	15,686,346	**A**/G	0.34	2.28E-06	8.40E-03	-0.31
Flour yield	*S1A_587581129*	1A	587,581,129	**A**/G	0.06	1.20E-05	3.05E-02	-0.48
Flour yield	*S1B_555294134*	1B	555,294,134	**C**/T	0.10	3.62E-06	1.20E-02	-0.48
Flour yield	*S1B_585326031*	1B	585,326,031	C/**G**	0.44	5.55E-07	3.22E-03	0.28
Flour yield	***S1B_653681752***	1B	653,681,752	**G**/T	0.14	5.38E-17	2.18E-12	-1.05
Flour yield	*S2A_57924183*	2A	57,924,183	**A**/G	0.42	9.10E-06	2.46E-02	-0.25
Flour yield	*S3A_621592864*	3A	621,592,864	**C**/T	0.31	7.74E-06	2.24E-02	-0.31
Flour yield	*S5A_382294123*	5A	382,294,123	G/**A**	0.24	1.37E-11	1.84E-07	0.55
Flour yield	*S5B_508665777*	5B	508,665,777	**T**/C	0.07	2.14E-07	1.45E-03	-0.64
Flour yield	***S6B_19335996***	6B	19,335,996	**G**/A	0.07	2.13E-13	4.32E-09	-0.89
Flour yield	*S6D_471614981*	6D	471,614,981	**T**/C	0.35	1.88E-07	1.45E-03	-0.32
Flour yield	*S7A_66283612*	7A	66,283,612	**A**/C	0.33	8.24E-07	4.17E-03	-0.34
Flour yield	*S7A_735818991*	7A	735,818,991	C/**G**	0.22	9.60E-07	4.32E-03	0.36
Flour yield	*S7B_123656516*	7B	123,656,516	**G**/C	0.15	7.25E-10	7.34E-06	-0.60
Flour ash	*S1D_20702150*	1D	20,702,150	**G**/T	0.23	2.54E-06	1.03E-02	0.01
Flour ash	*S1D_488805272*	1D	488,805,272	**G**/A	0.17	3.53E-06	1.30E-02	0.01
Flour ash	*S2A_77744254*	2A	77,744,254	G/**A**	0.09	1.18E-05	3.99E-02	-0.01
Flour ash	*S2B_794429290*	2B	794,429,290	C/**T**	0.30	5.61E-07	3.79E-03	-0.01
Flour ash	*S2D_551847796*	2D	551,847,796	G/**C**	0.10	2.19E-06	9.85E-03	-0.01
Flour ash	*S3D_540232458*	3D	540,232,458	C/**A**	0.08	1.60E-05	4.99E-02	-0.01
Flour ash	***S4A_120144412***	4A	120,144,412	T/**C**	0.10	4.63E-08	4.85E-04	-0.02
Flour ash	*S5A_3649534*	5A	3,649,534	**C**/A	0.33	3.13E-07	2.53E-03	0.01
Flour ash	*S5B_64633223*	5B	64,633,223	G/**A**	0.05	7.56E-07	3.83E-03	-0.02
Flour ash	*S5B_68052478*	5B	68,052,478	A/**T**	0.36	1.49E-09	3.02E-05	-0.01
Flour ash	*S5D_416596873*	5D	416,596,873	**T**/G	0.10	1.33E-10	5.38E-06	0.02
Flour ash	*S6D_469583807*	6D	469,583,807	**G**/C	0.10	7.00E-07	3.83E-03	0.01
Flour ash	*S7B_624947199*	7B	624,947,199	**C**/A	0.05	4.79E-08	4.85E-04	0.02
Milling score	***S1D_14707739***	1D	14,707,739	G/**T**	0.08	4.78E-08	3.88E-04	1.49
Milling score	*S2B_47200283*	2B	47,200,283	C/**G**	0.22	5.52E-06	2.24E-02	1.00
Milling score	*S2B_130028901*	2B	130,028,901	**G/**C	0.27	6.33E-06	2.33E-02	-0.59
Milling score	*S2B_759735490*	2B	759,735,490	T/**C**	0.27	3.51E-11	1.42E-06	1.01
Milling score	*S3B_82495634*	3B	82,495,634	G/**C**	0.07	1.64E-06	9.48E-03	1.17
Milling score	*S4A_724316409*	4A	724,316,409	G/**A**	0.27	1.67E-05	5.19E-02	0.58
Milling score	***S5A_20640566***	5A	20,640,566	**C**/T	0.09	1.41E-08	2.29E-04	-1.24
Milling score	*S5B_68052478*	5B	68,052,478	A/**T**	0.36	2.19E-06	1.11E-02	0.71
Milling score	*S5B_503326206*	5B	503,326,206	**C**/T	0.19	2.49E-06	1.12E-02	-0.78
Milling score	*S6B_27918221*	6B	27,918,221	C/**T**	0.24	5.81E-08	3.93E-04	0.90
Milling score	*S6D_471614981*	6D	47,161,4981	**T**/C	0.37	1.70E-08	2.29E-04	-0.83
Milling score	*S7D_319849357*	7D	319,849,357	G/**A**	0.14	4.16E-08	3.88E-04	1.25
Milling score	*SUN_31626104*	UN	31,626,104	G/**T**	0.25	1.08E-05	3.63E-02	0.65

^a^Significant single-nucleotide polymorphism (SNP). Markers in bold are markers with large effects

^b^Physical position of SNP sequence based on Wheat Chinese Spring IWGSC RefSeq v1.0

^c^SNP major allele/minor allele, the allele in bold is the favorable allele in soft white wheat (associated with higher phenotypic values for break flour yield, flour yield, and milling score and lower phenotypic values for flour ash)

^d^Minor allele frequency of the SNP

^e^*P* value of the significant SNP

^f^False discovery rate of the significant SNP

**Table 3:** Summary of SNP markers associated with flour functionality in 672 soft white winter wheat genotypes

Trait^a^	SNP^b^	Chromosome	Position (bp)^c^	Alleles^d^	MAF^e^	*P* value^f^	FDR^g^	Effect
Flour protein	***S1B_46883868***	1B	46,883,868	**C**/A	0.09	1.67E-09	3.37E-05	0.29
Flour protein	*S2A_159882069*	2A	159,882,069	**C**/T	0.09	4.24E-07	2.46E-03	0.24
Flour protein	*S2D_28763299*	2D	28,763,299	G/**A**	0.34	1.17E-08	1.19E-04	-0.18
Flour protein	*S3B_690878020*	3B	690,878,020	T/**C**	0.44	8.56E-09	1.16E-04	-0.19
Flour protein	*S4A_514430898*	4A	514,430,898	**C**/T	0.46	3.11E-12	1.26E-07	0.19
Flour protein	*S4B_63121316*	4B	63,121,316	**C**/T	0.10	2.21E-06	9.95E-03	0.22
Flour protein	*S5D_135645455*	5D	135,645,455	G/**A**	0.35	2.28E-08	1.85E-04	-0.17
Flour protein	*S7A_32805734*	7A	32,805,734	G/**A**	0.27	1.27E-06	6.46E-03	-0.16
Flour protein	*S7A_730416426*	7A	730,416,426	**G**/A	0.21	1.06E-05	4.00E-02	0.13
Flour protein	*S7D_507227865*	7D	507,227,865	G/**T**	0.05	1.09E-05	4.00E-02	-0.32
Flour protein	*SUN_351152321*	UN	351,152,321	A/**G**	0.31	1.35E-07	9.13E-04	-0.19
SDS sedimentation	*S1A_5326555*	1A	5326,555	G/**A**	0.30	1.59E-06	8.65E-03	-0.37
SDS sedimentation	*S1B_561712520*	1B	561,712,520	**G**/A	0.40	8.53E-14	3.45E-09	0.48
SDS sedimentation	***S1D_121990680***	1D	121,990,680	T/**G**	0.07	1.08E-06	7.26E-03	-0.67
SDS sedimentation	*S1D_223797230*	1D	223,797,230	C/**A**	0.05	1.78E-06	8.65E-03	-0.75
SDS sedimentation	***S1D_411063068***	1D	411,063,068	**T**/A	0.14	1.95E-10	3.95E-06	0.65
SDS sedimentation	*S2B_155110438*	2B	155,110,438	A/**G**	0.42	2.14E-06	8.65E-03	-0.33
SDS sedimentation	*S3A_697202279*	3A	697,202,279	**T**/C	0.09	7.40E-06	2.50E-02	0.47
SDS sedimentation	*S3B_723505334*	3B	723,505,334	T/**G**	0.43	1.18E-09	1.59E-05	-0.42
SDS sedimentation	*S5A_585018041*	5A	585,018,041	**A**/G	0.44	1.97E-06	8.65E-03	0.33
SDS sedimentation	*S5A_633105837*	5A	633,105,837	C/**A**	0.06	3.25E-06	1.20E-02	-0.75
SDS sedimentation	*S5B_539075483*	5B	539,075,483	**A**/G	0.11	7.34E-08	7.44E-04	0.61
SDS sedimentation	*S7A_108785365*	7A	108,785,365	**G**/A	0.07	1.06E-05	3.32E-02	0.93
SDS sedimentation	*S7A_675198728*	7A	675,198,728	C/**T**	0.11	1.42E-07	1.15E-03	-0.57
Water SRC	*S1B_547973154*	1B	547,973,154	**C**/T	0.10	5.59E-08	3.78E-04	0.60
Water SRC	***S1B_653681752***	1B	653,681,752	**G**/T	0.14	1.25E-10	5.08E-06	0.92
Water SRC	*S2A_613720768*	2A	613,720,768	T/**C**	0.06	2.02E-07	1.02E-03	-0.74
Water SRC	*S2B_66559534*	2B	66,559,534	**C**/A	0.08	5.49E-07	2.22E-03	0.69
Water SRC	*S3A_24993876*	3A	24,993,876	**C**/T	0.08	1.49E-07	8.61E-04	0.74
Water SRC	*S5A_382294123*	5A	382,294,123	G/**A**	0.24	2.88E-08	2.33E-04	-0.46
Water SRC	*S5A_673550305*	5A	673,550,305	C/**G**	0.37	1.18E-09	1.59E-05	-0.45
Water SRC	*S6B_29771821*	6B	29,771,821	G/**C**	0.35	2.97E-07	1.34E-03	-0.36
Water SRC	*S7A_709765148*	7A	709,765,148	**G**/T	0.12	9.56E-10	1.59E-05	0.69
Water SRC	*S7B_539196288*	7B	539,196,288	**A**/G	0.07	1.35E-08	1.37E-04	0.72
Water SRC	*S7D_327690580*	7D	327,690,580	G/**A**	0.26	8.76E-06	3.23E-02	-0.38
FSV	*S1A_534055653*	1A	534,055,653	G/**T**	0.05	1.70E-06	8.40E-03	0.74
FSV	*S1B_6678732*	1B	6,678,732	**A**/C	0.28	8.90E-09	3.60E-04	-0.38
FSV	*S2A_705583892*	2A	705,583,892	T/**C**	0.10	2.86E-06	9.65E-03	0.44
FSV	*S2B_7653964*	2B	7,653,964	C/**T**	0.19	2.28E-06	8.40E-03	0.43
FSV	*S2B_31382050*	2B	31,382,050	**G**/C	0.48	1.74E-06	8.40E-03	-0.27
FSV	*S3D_266839264*	3D	266,839,264	A/**G**	0.29	2.21E-06	8.40E-03	0.31
FSV	*S3D_601013637*	3D	601,013,637	A/**G**	0.13	4.77E-07	3.22E-03	0.49
FSV	*S4A_12168393*	4A	12,168,393	**C**/T	0.33	3.61E-07	3.22E-03	-0.33
FSV	*S4B_541252759*	4B	541,252,759	**A**/G	0.15	2.04E-06	8.40E-03	-0.44
FSV	*S5D_86308878*	5D	86,308,878	C/**A**	0.05	4.10E-08	8.31E-04	0.80
FSV	*S7A_200608114*	7A	20,060,8114	G/**T**	0.18	1.28E-05	3.71E-02	0.45
FSV	*S7A_583357214*	7A	583,357,214	G/**A**	0.05	4.20E-07	3.22E-03	0.73
FSV	*S7B_540056850*	7B	540,056,850	C/**T**	0.06	6.71E-08	9.06E-04	0.66
FSV	*S7D_38000037*	7D	38,000,037	**C**/T	0.06	5.62E-06	1.75E-02	-0.63

^a^Water SRC, water solvent retention capacity; FSV, flour swelling volume

^b^Significant single-nucleotide polymorphism (SNP). Markers in bold are markers with large effects

^c^Physical position of SNP sequence based on Wheat Chinese Spring IWGSC RefSeq v1.0

^d^SNP major allele/minor allele, the allele in bold is the favorable allele in soft white wheat (associated with higher phenotypic values FSV and lower phenotypic values for flour protein, SDS sedimentation, and water SRC)

^e^Minor allele frequency of the SNP

^f^*P* value of the significant SNP

^g^False discovery rate of the significant SNP

**Table 4:** Summary of SNP markers associated with cookie diameter in 672 soft white winter wheat genotypes

Trait	SNP^a^	Chromosome	Position (bp)^b^	Alleles^c^	MAF^d^	*P* value^e^	FDR^f^	Effect
Cookie diameter	*S1A_586706397*	1A	586,706,397	**G**/T	0.06	1.89E-09	3.83E-05	-0.08
Cookie diameter	*S1B_573323546*	1B	573,323,546	**G**/A	0.12	6.74E-06	2.62E-02	-0.06
Cookie diameter	*S2D_28832058*	2D	28,832,058	T/**C**	0.40	2.25E-06	1.01E-02	0.04
Cookie diameter	*S4A_583968823*	4A	583,968,823	G/**A**	0.10	8.52E-08	5.77E-04	0.06
Cookie diameter	*S4A_688407511*	4A	688,407,511	**G**/A	0.17	5.03E-08	5.09E-04	-0.05
Cookie diameter	*S4B_667833352*	4B	667,833,352	**C**/T	0.18	1.33E-06	6.73E-03	-0.06
Cookie diameter	*S5A_555334864*	5A	555,334,864	C/**T**	0.43	1.27E-09	3.83E-05	0.05
Cookie diameter	*S5B_418463680*	5B	418,463,680	**A**/T	0.18	9.94E-08	5.77E-04	-0.06
Cookie diameter	*S5B_571725191*	5B	571,725,191	T/**A**	0.36	7.11E-06	2.62E-02	0.03
Cookie diameter	*S5B_580976632*	5B	580,976,632	A/**C**	0.18	1.25E-05	3.89E-02	0.04
Cookie diameter	*S7B_648784573*	7B	648,784,573	G/**A**	0.10	4.48E-08	5.09E-04	0.07
Cookie diameter	*S7D_56065854*	7D	56,065,854	**C**/G	0.18	9.97E-08	5.77E-04	-0.05
Cookie diameter	*SUN_82579190*	UN	82,579,190	**G**/C	0.14	9.93E-06	3.35E-02	-0.05

^a^Significant single-nucleotide polymorphism (SNP). Markers in bold are markers with large effects

^b^Physical position of SNP sequence based on Wheat Chinese Spring IWGSC RefSeq v1.0

^c^SNP major allele/minor allele, the allele in bold is the favorable allele in soft white wheat (associated with higher cookie diameter)

^d^Minor allele frequency of the SNP

^e^*P* value of the significant SNP

^f^False discovery rate of the significant SNP

### Marker-trait associations

Based on the LD between markers, each MTA identified from the FarmCPU models represent a distinct locus or QTL. Considering all traits together, a total of 178 significant MTAs were identified across all wheat chromosomes (Tables 1,2,3,4). Sixty-two MTAs were detected on the A genome, 77 MTAs on the B genome, 34 MTAs on the D genome, and five MTAs on unaligned (UN) chromosomes. Chromosome 1B and 7A carried the highest number of MTAs (*n* = 16), whereas chromosome 4D had only a single MTA. The favorable alleles and their corresponding frequencies are described in Tables 1,2,3,4. There were 12 large-effect markers associated with 11 traits (1–2 markers per trait) (Table 5). For SKCS size, FSV, and cookie diameter, all significant markers had small effects.

**Table 5:** Allelic effect of large-effect markers on end-use quality traits in soft white wheat

Trait	Markers	MAF^a^	Alleles^b^	N^c^	Mean BLUE^d^	Tukey's test^e^
SKCS hardness	*S5A_480515221*	0.14	CC	572	25.6	A
			CT	18	20.6	B
			**TT**	82	18.9	B
	*S6B_705613777*	0.08	AA	13	32.8	A
			AG	75	24.9	B
			**GG**	584	24.5	B
SKCS weight	*S2B_533178165*	0.09	**CC**	569	39.3	A
			CG	89	37.9	B
			GG	14	35.8	B
Test weight	*S4B_413497949*	0.09	**CC**	602	62.1	A
			CT	15	61.3	AB
			TT	55	60.9	B
Grain protein	*S1B_46883868*	0.09	AA	46	11.9	A
			AC	17	11.8	A
			**CC**	609	11.1	B
Break flour yield	*S1B_653681752*	0.14	**GG**	494	48.3	A
			GT	171	47.3	B
			TT	7	45.5	C
Flour yield	*S1B_653681752*	0.14	**GG**	494	69.6	A
			GT	171	68.7	B
			TT	7	66.9	B
	*S6B_19335996*	0.07	AA	38	67.0	B
			AG	16	68.1	B
			**GG**	618	69.5	A
Flour ash	*S4A_120144412*	0.10	**CC**	49	0.36	B
			CT	29	0.36	B
			TT	594	0.39	A
Milling score	*S1D_14707739*	0.08	**GG**	556	84.7	B
			GT	84	86.8	A
			TT	10	88.5	A
	*S5A_20640566*	0.09	**CC**	576	85.2	A
			CT	31	85.7	A
			TT	43	82.8	B
Flour protein	*S1B_46883868*	0.09	**CC**	609	9.2	B
			CA	17	9.7	A
			AA	46	9.8	A
SDS Sedimentation	*S1D_121990680*	0.07	**GG**	16	9.4	B
			GT	58	10.2	AB
			TT	598	10.7	A
	*S1D_411063068*	0.14	AA	76	11.9	A
			AT	25	10.8	AB
			**TT**	571	10.5	B
Water SRC	*S1B_653681752*	0.14	**GG**	494	54.0	C
			GT	171	55.1	B
			TT	7	58.9	A

^a^Minor allele frequency

^b^Allele in bold is the favorable allele in soft white wheat

^c^Number of genotypes per marker allele

^d^BLUE, best linear unbiased estimators

^e^The letters represent results of Tukey’s HSD test (treatments with different letters are significantly different at 95% level of confidence)

For grain characteristics, five MTAs with large effects were detected on chromosome 1B, 2B, 4B, 5A, and 6B (Table 5). Markers *S5A_480515221* and *S6B_705613777* were associated with SKCS hardness and impacted the hardness index by 6.7 and 7.9 units on average, respectively. Marker *S2B_533178165* was associated with SKCS weight and influenced the phenotype by 3.55 mg. For test weight, *S4B_413497949* had the largest effect and resulted in 1.2 kg/hL difference in the phenotype on average, whereas for grain protein content, *S1B_46883868* had the largest effect with 0.85% increase/decrease in the phenotype. *S1B_46883868* was also associated with flour protein content and affected the trait value by 0.63% on average.

For milling traits, five markers had large effects and were detected on chromosomes 1B, 1D, 5A, and 6B (Table 5). Marker *S1B_653681752* was associated with break flour yield and flour yield and influenced trait values by 2.9% and 2.7% on average, respectively. An additional large effect marker was associated with flour yield: *S6B_19335996* affected the trait value by 2.5%. Marker *S4A_120144412* was associated with flour ash and influenced the phenotype by 0.03% on average. For milling score, *S1D_14707739* and *S5A_20640566* affected the phenotype by 3.8 and 2.4 units, respectively.

For flour parameters, there were four markers with large effects located on chromosomes 1B, 1D, and 4A (Table 5). In addition to break flour yield, *S1B_653681752* also influenced water SRC by 4.9% on average. For SDS sedimentation, two large effect markers were identified including *S1D_121990680* and *S1D_411063068*, which affected the trait values by 1.3 and 1.4 g/mL, respectively*.* Except for *S5A_480515221*, *S4A_120144412*, and *S1D_121990680*, which were associated with SKCS hardness, flour ash, and SDS sedimentation, the favorable alleles for the remaining nine large effect markers were present in high frequencies (86–93%) in this soft white wheat germplasm.

### Loci associated with at least two end-use quality traits

Among the 178 MTAs identified in this study, there were 17 loci associated with more than a single end-use quality trait (Table 6). Among these loci, there were two large-effect markers (*S1B_46883868* and *S1B_653681752*) and ten small-effect markers that were associated with at least two end-use quality traits. For each of these 12 markers, there was desirable linkage between the favorable alleles. This suggests that these markers are having desirable pleiotropic effects and could be useful to simultaneously breed for more than a single end-use quality trait. Based on pairwise LD estimates between physically close markers, there were additionally five loci on chromosomes 1A, 1B, 6B, 7A, and 7B that were associated with more than a single end-use quality trait (Table 6). For each of these loci, LD between significant markers that were associated with different traits was higher than the *r*^*2*^ threshold of 0.1. For three of these loci, *S1A_586706397*/*S1A_587581129*, *S6B_27918221/ S6B_29771821* and *S7A_730416426/S7A_731026067*, there was desirable linkage between marker alleles in each locus, whereas for the other two loci *S1B_561712520*/*S1B_569507932* and *S7B_624947199/S7B_636744313*, there was unfavorable linkage. Therefore, for the latter two loci, selecting for one trait could negatively affect the other trait.

**Table 6:** Loci associated with two or more end-use quality traits in 672 soft white winter wheat genotypes

Markers^a^	SKCS hardness	SKCS size	SKCS weight	Test weight	Grain protein	Break flour yield	Flour yield	Flour ash	Milling Score	Flour protein	SDS sedimentation	Water SRC	Cookie diameter
*S1A_586706397*/*S1A_587581129*							** × **						** × **
***S1B_46883868***					** × **					** × **			
*S1B_561712520*/*S1B_569507932*		** × **									** × **		
***S1B_653681752***						** × **	** × **					** × **	
*S2D_563799166*		** × **	** × **										
*S3B_690878020*					** × **					** × **			
*S4B_63121316*					** × **					** × **			
*S5A_382294123*							** × **					** × **	
*S5B_68052478*								** × **	** × **				
*S5B_508665777*						** × **	** × **						
*S6B_27918221/ S6B_29771821*									** × **			** × **	
*S6B_583281710*		** × **	** × **										
*S6D_471614981*							** × **		** × **				
*S7A_730416426*/*S7A_731026067*					** × **					** × **			
*S7B_624947199*/*S7B_636744313*				** × **				** × **					
*S7D_56065854*	** × **					** × **							** × **
*SUN_351152321*					** × **					** × **			

^a^Markers in bold had large effects. Markers before and after the forward slash (/) were in linkage disequilibrium (*r*^*2*^ ≥ 0.1). *S1A_586706397* and *S1A_587581129* were associated with cookie diameter and flour yield, respectively, *S1B_561712520* and *S1B_569507932* were associated with SDS sedimentation and SKCS size, respectively, *S6B_27918221* and *S6B_29771821* were associated with milling score and water SRC, respectively. *S7A_730416426* and *S7A_731026067* were associated with flour protein and grain protein content, respectively, and *S7B_624947199* and *S7B_636744313* were associated with flour ash and test weight, respectively

### Co-localized MTAs with previously identified end-use quality genes/QTL

A total of 35 annotated genes were located close to the physical positions of the 12 large effect markers. The putative functions of these genes are described in Supplementary Table S3. In addition, we found 13 GWAS MTAs available at the IWGSC sequence repository that were within the genomic regions of the 12 large effect markers identified in our study. These GWAS MTAs from previous studies were associated with thousand kernel weight, test weight, grain fill duration, grain protein content, SDS sedimentation, and grain minerals (Cu and Zn) (Supplementary Table S3). Furthermore, comparative mapping (based on physical positions of molecular markers) between all the 178 identified MTAs from this study and end-use quality QTL/genes from previous genetic studies [2, 4, 6–17, 19–22] showed that 40 MTAs were positioned within genomic regions of previously discovered end-use quality genes/QTL (Supplementary Table S4).

Of the 16 identified loci for SKCS hardness in this study, 10 loci were found within genomic regions of previously reported grain hardness QTL (Supplementary Table S4). For instance, SKCS hardness associated markers in this study, *S1A_583894689*, *S3A_690786387*, *S3B_718850970*, *S5A_480515221*, *S6B_130163859*, *S6B_705613777*, and *S7A_12011069* were located close to the positions of previously reported grain hardness associated markers/QTL, *QGh.caas-1A* (~ 575 Mb, [9]), *wPt-4725* (709 Mb [12],), *QKh.WJ-3B.3* (~ 695 Mb, [8]), *S5A_463766631* (464 Mb, [22]), *IWB11485* (121 Mb, [2]), *S6B_703822990* (704 Mb, [22]), *wPt-0744* (0.2 Mb, [12]), respectively. Similarly, *S1B_635869100* was positioned 8–13 Mb from *Qgh-1B* [13], *QKh.WY-1B.1b* [8], *QGh.cass-1B* [7], *QNhi.hwwgr-1BL* [10], and *QKH.ksw-1B* [21]. On chromosome 5B, *S5B_549556474* identified in this study was found close to *Qshi.hwwgr-5BL* (566–571 Mb, [10]) and a QTL flanked by the SSR marker *wmc289* (556 Mb, [11]). In addition, a MTA on chromosome 7B, *S7B_643501407*, was located at ~ 8 Mb from *QKh.WJ-7B.1B* [8] and *QGh.caas-7B.1b* [7]. Our comparative mapping showed the importance of these 10 previously characterized loci in controlling kernel hardness and suggests that the remaining six loci could be novel.

Three loci associated with SKCS size were located close to previously identified kernel size QTL (Supplementary Table S4). For instance, *S2B_154846350*, associated with SKCS size in this study, was located at 4–5 Mb from *IWB30179* [2] and *QKd.cob-1A* (~ 134 Mb, [14]) that were associated with SKCS size and kernel diameter, respectively. Similarly, *S2D_563799166* and *S5A_578074731* were close to previously identified kernel diameter QTL, *QKd.hwwgr-2DL* (~ 552 Mb, [10]) and *QKD.ksu-5A1* (568 Mb, [21]), respectively. For SKCS weight, *S2D_563799166* identified in this study was positioned at ~ 12 Mb from *QSkw.hwwgr-2DL* (552 Mb, [10]) (Supplementary Table S4). Two of our identified MTAs for test weight were found near previously identified test weight associated markers/QTL. This includes *S2A_762292662* located close to *IWB35564* (760 Mb, [2]) and *S7B_40194878* located at 7–8 Mb from *QTW.ksu-7B* [21] and *IWB54370* [2] (Supplementary Table S4).

Four of the associated loci with grain protein content in this study were previously reported (Supplementary Table S4). This includes *S1B_633974958*, which was positioned close to *QGpc.caas-1B.1* (628–638 Mb) and *QWgc.caas-1B* (~ 628–634 Mb), which were associated with grain protein content and wet gluten content, respectively [7]. Similarly, *S4B_63121316* and *S5D_543253602* were found close to associated markers with grain protein content *gwm368* (60 Mb, [11]) and *wPt-9788*/*wPt-0400* (560 Mb, [12]), respectively. On chromosome 7A, the grain protein content associated marker, *S7A_731026067* was 18 Mb from *Qgpc.7A.1* (*wmc525*, [19]), which was associated with both protein content and dry gluten content**.**

For break flour yield, the associated locus tagged by marker *S3B_630394456* was at the physical position of *IWA6254* (630 Mb) also associated with break flour yield [17]. For flour yield, five of the identified MTAs were mapped close to flour yield associated loci in previous genetic studies (Supplementary Table S4). For instance, *S1A_9128313* and *S1A_15686346* were in close proximity to *QFY.ksu-1A* (7 Mb, [21]). *S1A_9128313* and *S1A_15686346* were also within the genomic regions of the genes *TraesCS1A01G010900* (5 Mb) and *TraesCS1A01G039600* (16 Mb), respectively. *TraesCS1A01G010900* (5 Mb) and *TraesCS1A01G039600* were annotated as low molecular weight glutenin subunit and high molecular weight glutenin subunit, respectively. Another MTA on chromosome 1B associated with flour yield, *S1B_653681752*, was identified close to *QFY.ksu-1B* (649 Mb) [21]. Similarly, *S5A_382294123* and *S6B_19335996* were found in proximity to *IWB76667* (384 Mb, [2]) and *IWA7725* (27 Mb, [17]), respectively. Two flour ash associated loci in this study, *S5A_3649534* and *S5B_68052478*, were positioned close to *Qgac.cob-5A* (*gwm443*, 11 Mb) and *Qgac.cob-5B.1* (*gwm540*, 67 Mb), respectively, which were previously identified by El-Feki et al. [14] (Supplementary Table S4).

For flour protein, *S2D_28763299* was located 15–17 Mb from *QGpc.caas-2D* [7], which was associated with grain protein content and *QWgc.WY-2D.5* [8], which was associated with wet gluten content. Similarly, *S7A_730416426* associated with flour protein content in this study was ~ 17 Mb away from *qGPC.7A.1* [19], which was associated with grain protein content and dry gluten content (Supplementary Table S4).

Two SDS sedimentation associated markers in this study were previously identified (Supplementary Table S4). These loci include *S1B_561712520* located close to *IWB14950* (558 Mb, [2]) and the gene *Glu-B1* (556 Mb) and *S5B_539075483*, which mapped close to *QSsd.caas-5B* (527 Mb, [9]). Among water SRC associated markers, *S1B_547973154*, *S1B_653681752*, *S2B_66559534*, and *S6B_29771821* were positioned near *Glu-B1* (556 Mb), *IWB27057* (652 Mb, [2]), *IWA820* (44 Mb, [17]), and *IWA7725* (27 Mb, [17]), respectively (Supplementary Table S4).

## Discussion

This study used historical data that captured a wide range of phenotypic variation for end-use quality within a soft white winter wheat breeding program. Heritability estimates for end-use quality traits were moderate to high except for grain and flour protein content. This suggests that most traits are primarily controlled by genetic factors and that a genotypic selection (as opposed to phenotypic selection) is a rational strategy. This study identified the genetic architecture underlying 14 end-use quality traits among recent breeding lines and cultivars from a soft white winter wheat breeding program. Prior to this study, Jernigan et al. [2] investigated the genetic architecture of end use quality in a set of 480 advanced soft white winter wheat breeding lines and cultivars from Pacific Northwest breeding programs selected from 1992 to 2014. Thus, the germplasm used in this study is different from that used by Jernigan et al. [2]. Consequently, our investigation was expected to corroborate previous and/or discover additional QTL associated with end-use quality in soft white wheat.

Identified MTAs in this study as well as genotypes with favorable alleles will be useful for end-use quality improvement in soft white and other types of wheat. The 12 large effect markers can be converted into Kompetitive Allele Specific PCR (KASP) or thermal asymmetric reverse PCR (STARP) markers for use in marker-assisted selection (MAS). Among these large effect markers, *S1B_653681752* is useful to breed for higher break flour yield and flour yield and lower water SRC. Similarly, *S1B_46883868* is associated with both grain protein content and flour protein. The favorable alleles of nine of the large effect markers were present in high frequencies in this germplasm. This suggested that these markers were under high selection pressure in the soft white wheat breeding program, likely the result of long-term phenotyping and selection, and the pyramiding of favorable alleles across the breeding populations. Based on our comparative mapping, eight of the large effect markers including *S1B_46883868*, *S1D_14707739*, *S1D_121990680*, *S1D_411063068*, *S2B_533178165*, *S4A_120144412*, *S4B_413497949*, and *S5A_20640566* were not reported in previous studies, thus should be prioritized for MAS. Only a few loci were found to have large effects, suggesting that many end-use quality traits have complex genetic architecture and are mainly controlled by several minor genes with small effects. For some traits like SKCS diameter, FSV, and cookie diameter, all identified markers had small effects, suggesting that MAS may not be useful for these traits. Therefore, genomic selection might be a better approach to implement for such traits [27, 55].

### Grain characteristics

Grain characteristics greatly influence wheat end-use quality [4, 7, 11, 30, 47]. Grain hardness affects most end-use quality traits including break flour yield, flour yield, flour particle size, starch damage, dough strength, and cookie diameter [27, 56–58]. The variation in grain hardness in the present soft wheat germplasm, like most soft wheat breeding populations, is independent of the puroindolines because wild-type puroindoline genes at the *Ha* locus are generally fixed. This is consistent that no MTAs were identified on chromosome 5DS in this study. Other grain characteristics including SKCS size, SKCS weight, test weight, and grain protein influence wheat milling performance [28, 30]. SKCS size and SKCS weight were highly correlated in this germplasm (*r* = 0.8; [27]) and this was reflected in the GWAS in which *S2D_563799166* and *S6B_583281710* were found to be associated with both traits.

Grain protein content is an essential quality trait that affects flour functionality. Unlike bread, soft wheat products often require lower protein levels to minimize gluten formation and mixing strength [5]. The positive correlation (*r* = 0.4–05; [27]) between grain/flour protein content and SDS sedimentation (a measure of gluten strength) in this germplasm provides further evidence of their direct relationship. However, based on the GWAS, no significant markers were in common between SDS sedimentation and grain/flour protein content. Grain and flour protein were phenotypically correlated in this germplasm [27]. This relationship was also evident in our GWAS in which five markers were associated with both grain and flour protein. Grain and flour protein in this wheat collection had low heritability estimates and high genotype by environment interactions as described by Aoun et al. [27]. Consequently, most markers associated with grain/flour protein in this study had small effects, except for marker *S1B_46883868*.

### Milling traits

Higher break flour yield, flour yield, lower flour ash, and higher milling score are desirable traits in soft wheat. Cultivars with alleles that increase these traits could lead to higher milling performance and thus greater profit for flour millers. Moderate to high heritability estimates and positive correlations among milling traits in this germplasm [27] suggest that genetic gain and simultaneous breeding for these traits is possible. Positive correlations between milling traits were also obvious in our GWAS results. For instance, *S1B_653681752* and *S5B_508665777* favorable alleles for break flour yield were also associated with higher flour yield. Similarly, *S6D_471614981*, a favorable allele for flour yield was also associated with higher milling score. Negative correlations between milling score and ash in this germplasm (*r* = -0.7) were discussed in Aoun et al. [27]. This desirable negative correlation was also reflected in our GWAS in which the *S5B_68052478* minor allele was associated with lower ash and higher milling score. We found that *S1B_100055026*, which was associated with break flour yield, was located close to *Glu-B3* gene flanked by the DArT marker *wPt-1317* (137 Mb, [14]). Similarly, the flour yield associated marker in this study, *S1B_555294134*, was located 1 Mb from *Glu-B1* (556 Mb). It is well known that glutenin subunit families are major components of wheat endosperm storage proteins and are associated with many end-use quality traits. The presence of break flour yield and flour yield associated loci close to *Glu-B1* and *Glu-B3* may suggest that there is a genetic association between endosperm storage proteins and endosperm structure as evidenced by Boehm Jr et al. [59]. The composition of the protein matrix surrounding starch granules likely contributes to the mechanical strength of the endosperm.

### Flour and baking parameters

Unlike bread, confectionary products require lower gluten strength and water absorption capacity, which were measured using SDS sedimentation and water SRC, respectively. Higher water SRC is in part due to starch damage from milling and non-starch polysaccharides [5, 33, 60] and thus, lower water absorption is preferred as it results in better cookie spread and lower viscosity batters. Three water SRC associated markers co-localized with milling trait associated markers including *S1B_653681752*, *S5A_382294123*, and *S6B_27918221/ S6B_29771821*. Negative correlations between water SRC and milling traits previously discussed by Aoun et al. [27] were also observed in our GWAS results particularly for markers *S1B_653681752* and *S5A_382294123*.

Higher FSV is desirable for making some Asian-style noodles [1, 36]. We found that *S1A_534055653*, which was associated with FSV in our study was near the gene *Glu-A1* flanked by the SSR marker *wmc312* (511 Mb, [14]). This result suggests genetic correlation between gluten content/strength and FSV. Similar observation was also found for cookie diameter in which its associated marker *S1B_573323546* was close to the position of the gene *Glu-B1.* The FSV associated marker *S7D_38000037* from this study was 2 Mb from the waxy locus *Wx-D1*. The association between *S7D_38000037* and any null allele at *Wx-D1* is at present unknown, but is unlikely as the known *Waxy* allele at *Wx-D1* is rare [61]*.* Similarly, we did not identify MTAs for FSV that were close to the locations of the other homoeologous waxy loci *Wx-A1* and *Wx-B1* which were located on chromosome 7A and 4A, respectively [35, 62]. Mutation/deletion in any of the three waxy loci often results in reduced amylose ‘partial waxy’ wheat which is associated with higher FSV. Therefore, the variation in FSV in this germplasm is likely independent of the waxy loci. As noted above, there were no major QTL identified for cookie baking. As such, alternative genotypic selection strategies such as genomic selection may be more appropriate for this trait.

## Conclusion

In this study we investigated the phenotypic and genotypic structure of 14 end-use quality traits in 672 soft white winter wheat breeding lines and cultivars adapted to the Pacific Northwest region of the United States. A total of 178 MTAs were identified across all wheat chromosomes of which 40 MTAs were positioned within genomic regions of previously discovered end-use quality genes/QTL. These results highlight the fact that among the multitude of traits that a wheat breeder selects for, end-use quality is a relatively large proportion. The high heritability of most traits underscores the success of long-term phenotypic selection. Among the identified MTAs, 12 markers had large effects (eight of them were previously uncharacterized) and thus could be prioritized in breeding programs. For example, a relatively manageable number of lines, say, those resulting from head row selection, could be subjected to a single round of genotypic selection to fix the favorable allele at one or more of the large effect loci. Such a strategy could return benefits later on as a greater proportion of lines would meet end-use quality targets during subsequent replicated yield trials. This study also revealed that for some end-use quality traits (SKCS size, FSV, and cookie diameter), only small effect markers were identified, suggesting that these traits are controlled by multiple minor genes in this germplasm, and that alternative selection strategies such as genomic selection could augment traditional and laborious phenotyping.

## Supplementary Information

**Additional file 1: Supplementary Fig. S1. **Principal component(PC) analysis obtained from 40,518 SNPs in 672 soft white winter wheat genotypes. The first two PCs, PC1 and PC2 explaining 5.3% and 4.0% of the variation, respectively. **Supplementary Fig. S2**. Scatter plot representing the genome-wise linkage disequilibrium (LD) decay. The LD estimate (*r*^2^)for pairs of SNPs was plotted against the corresponding physical positions inmega base pairs (Mb) based on IWGSC Wheat Chinese Spring IWGSC RefSeq v1.0. The dashed red line represents the population-specific critical value of *r*^2^=0.1.**Supplementary Fig. S3. **Scatter plot representing the chromosome-wise linkage disequilibrium (LD) decay. The LD estimate (*r*^2^) for pairs of SNPs was plotted against the corresponding physical positions in megabase pairs (Mb) based on Wheat Chinese Spring IWGSC RefSeq v1.0. The dashed redline represents the LD population threshold of 0.1. **Supplementary Fig. S4. **Quantile-Quantile plots of the expected -log_10_(*P*) versus the observed -log_10_(*P*) for association mapping model MLM for the14 end-use quality traits. **Supplementary Fig. S5.** Quantile-Quantile plots of the expected -log_10_ (*P*) versus the observed -log_10_(*P*) for association mapping model FarmCPU for the 14 end-use quality traits. **Supplementary Fig. S6.** Quantile-Quantile plots of the expected-log_10_ (*P*) versus the observed -log_10_(*P*) for association mapping model BLINK for the 14 end-use quality traits. **Supplementary Fig. S7.** Summary of genome-wide association studies for 14 end-use quality traits in 672 soft winter wheat genotypes based on Fixed and random model Circulating Probability Unification model. The horizontal red line indicates significance level at FDR ≤ 0.05.

**Additional file 2: Table S1.** Summary of GWAS for 14 end-use quality in softwhite winter wheat using mixed linear model (MLM). **Table S2.** Summary of GWAS for 14 end-use quality in soft whitewinter wheat using Bayesian-information and Linkage-disequilibrium Iteratively Nested Keyway (BLINK) model. **Table S3. **Annotated genes within the genomic regions of large-effect markers identified in this study for end-use quality traits in soft white winter wheat. **Table S4.** Previously identified QTL within the genomic regions of Marker-Trait-Associations identified in this study.

## Data Availability

The datasets generated and analyzed during the current study are available in the T3/wheat repository, https:/wheat.triticeaetoolbox.org/.
