# Supplementary material for: Genetic architecture of end-use quality traits in soft white winter wheat
Source: BMC Genomics. 2022 Jun 14;23:440. doi: 10.1186/s12864-022-08676-5 (PMC9195237; doi:10.1186/s12864-022-08676-5)
Supplement: Supplementary file 1 — Additional file 1: Supplementary Fig. S1. Principal component(PC) analysis obtained from 40,518 SNPs in 672 soft white winter wheat genotypes. The first two PCs, PC1 and PC2 explaining 5.3% and 4.0% of the variation, respectively. Supplementary Fig. S2. Scatter plot representing the genome-wise linkage disequilibrium (LD) decay. The LD estimate (r2)for pairs of SNPs was plotted against the corresponding physical positions inmega base pairs (Mb) based on IWGSC Wheat Chinese Spring IWGSC RefSeq v1.0. The dashed red line represents the population-specific critical value of r2=0.1.Supplementary Fig. S3. Scatter plot representing the chromosome-wise linkage disequilibrium (LD) decay. The LD estimate (r2) for pairs of SNPs was plotted against the corresponding physical positions in megabase pairs (Mb) based on Wheat Chinese Spring IWGSC RefSeq v1.0. The dashed redline represents the LD population threshold of 0.1. Supplementary Fig. S4. Quantile-Quantile plots of the expected -log10(P) versus the observed -log10(P) for association mapping model MLM for the14 end-use quality traits. Supplementary Fig. S5. Quantile-Quantile plots of the expected -log10 (P) versus the observed -log10(P) for association mapping model FarmCPU for the 14 end-use quality traits. Supplementary Fig. S6. Quantile-Quantile plots of the expected-log10 (P) versus the observed -log10(P) for association mapping model BLINK for the 14 end-use quality traits. Supplementary Fig. S7. Summary of genome-wide association studies for 14 end-use quality traits in 672 soft winter wheat genotypes based on Fixed and random model Circulating Probability Unification model. The horizontal red line indicates significance level at FDR ≤ 0.05. [file 12864_2022_8676_MOESM1_ESM.docx]

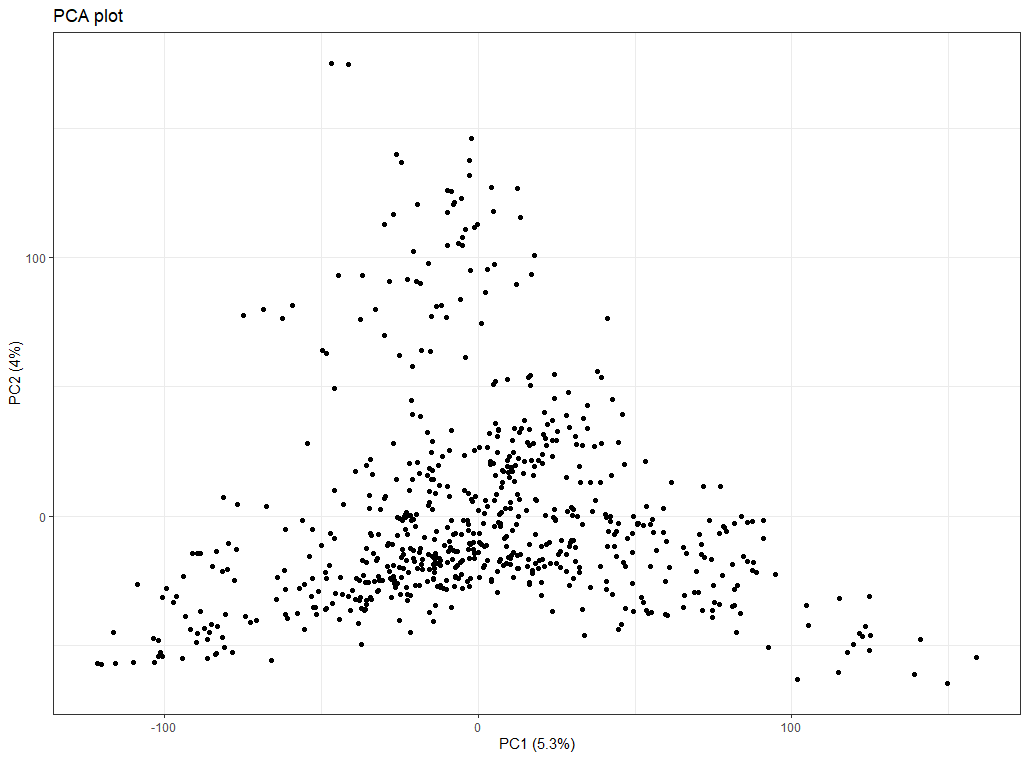
**Supplementary Fig. S1.** Principal component (PC) analysis obtained from 40,518 SNPs in 672 soft white winter wheat genotypes. The first two PCs, PC1 and PC2 explaining 5.3% and 4.0% of the variation, respectively.


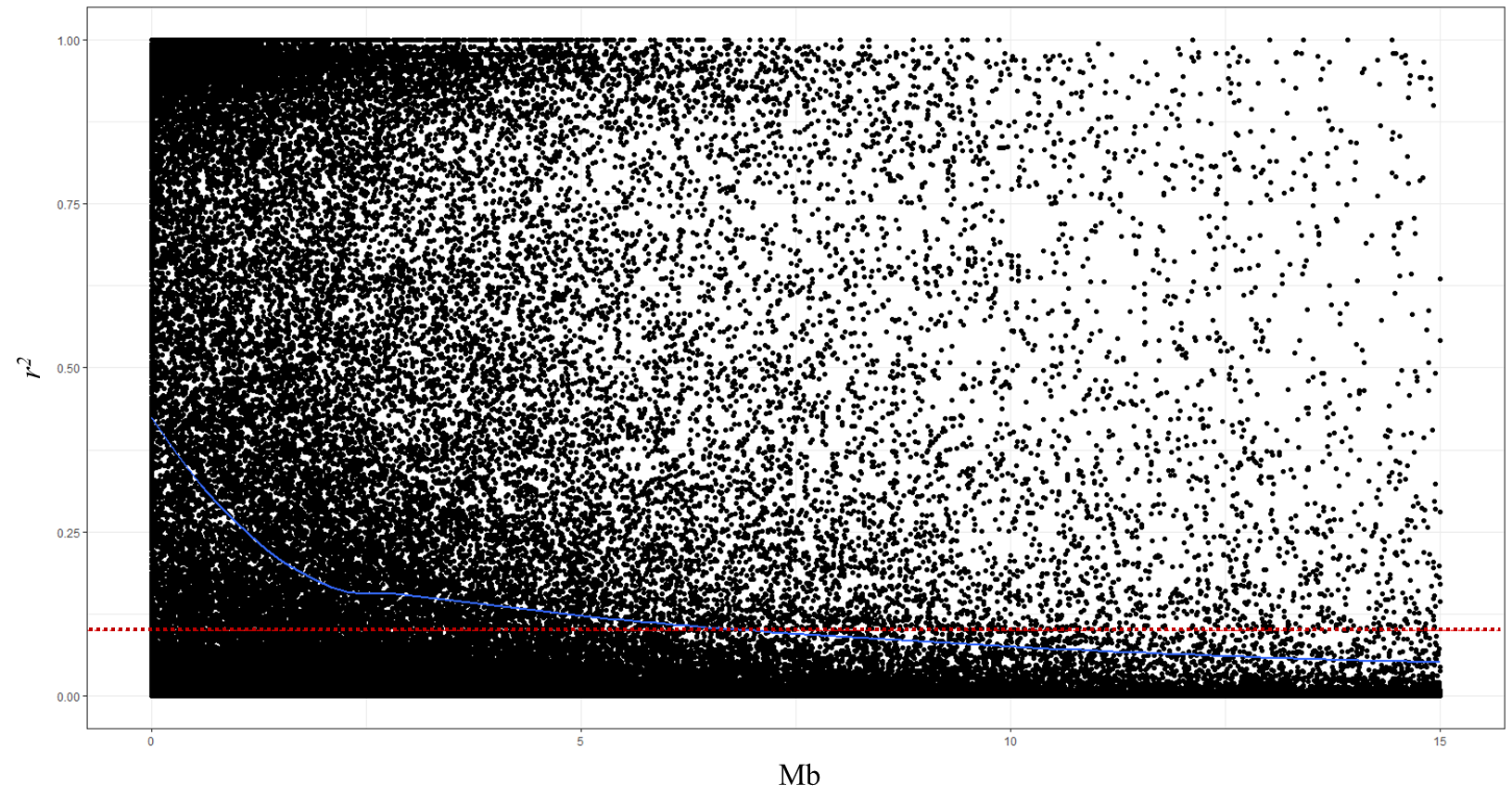
 **Supplementary Fig. S2**. Scatter plot representing the genome-wise linkage disequilibrium (LD) decay. The LD estimate (*r^2^*) for pairs of SNPs was plotted against the corresponding physical positions in mega base pairs (Mb) based on IWGSC Wheat Chinese Spring IWGSC RefSeq v1.0. The dashed red line represents the population-specific critical value of *r^2^*=0.1


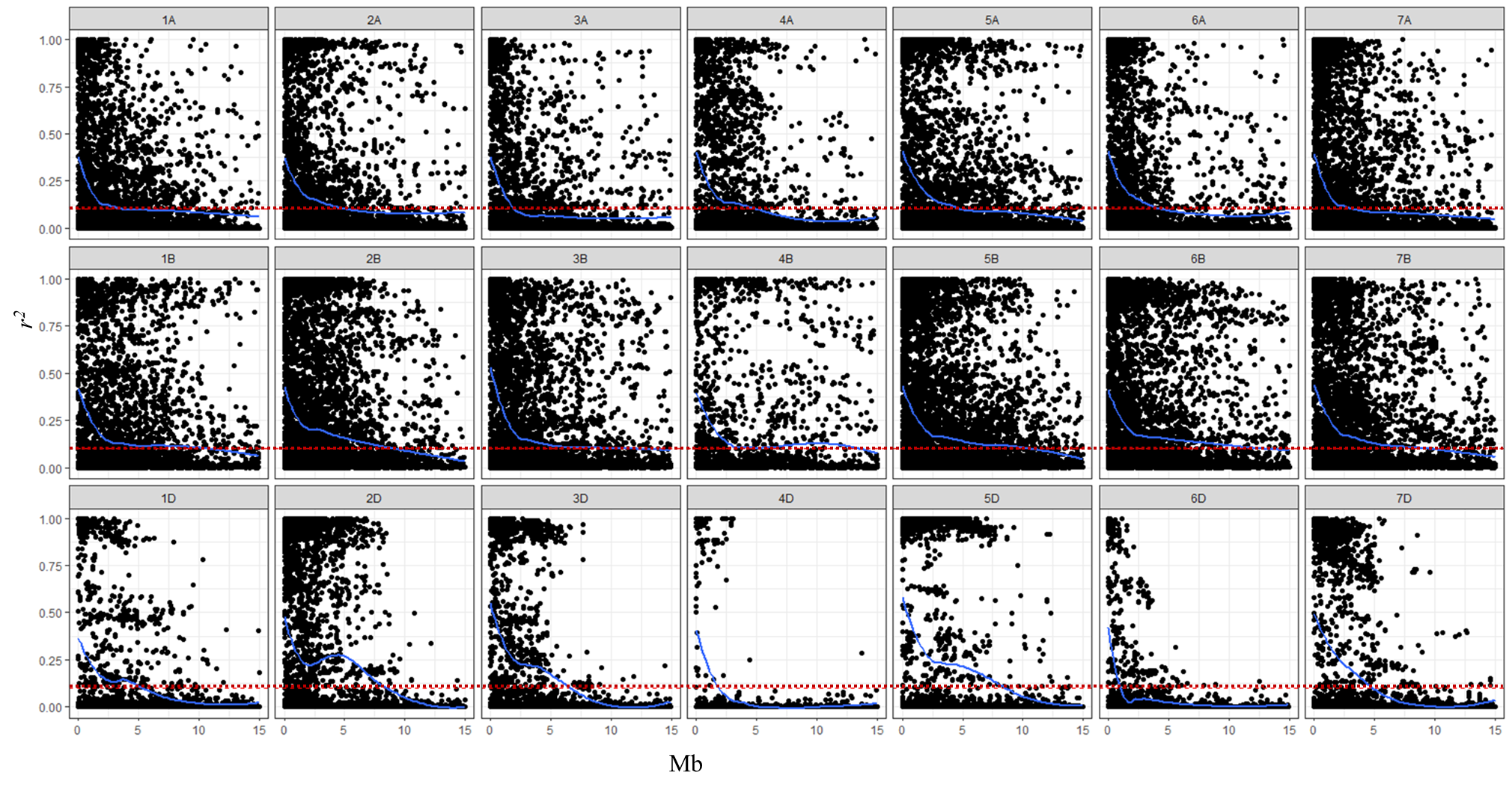
**Supplementary Fig.S3.** Scatter plot representing the chromosome-wise linkage disequilibrium (LD) decay. The LD estimate (*r^2^*) for pairs of SNPs was plotted against the corresponding physical positions in mega base pairs (Mb) based on Wheat Chinese Spring IWGSC RefSeq v1.0. The dashed red line represents the LD population threshold of 0.1.


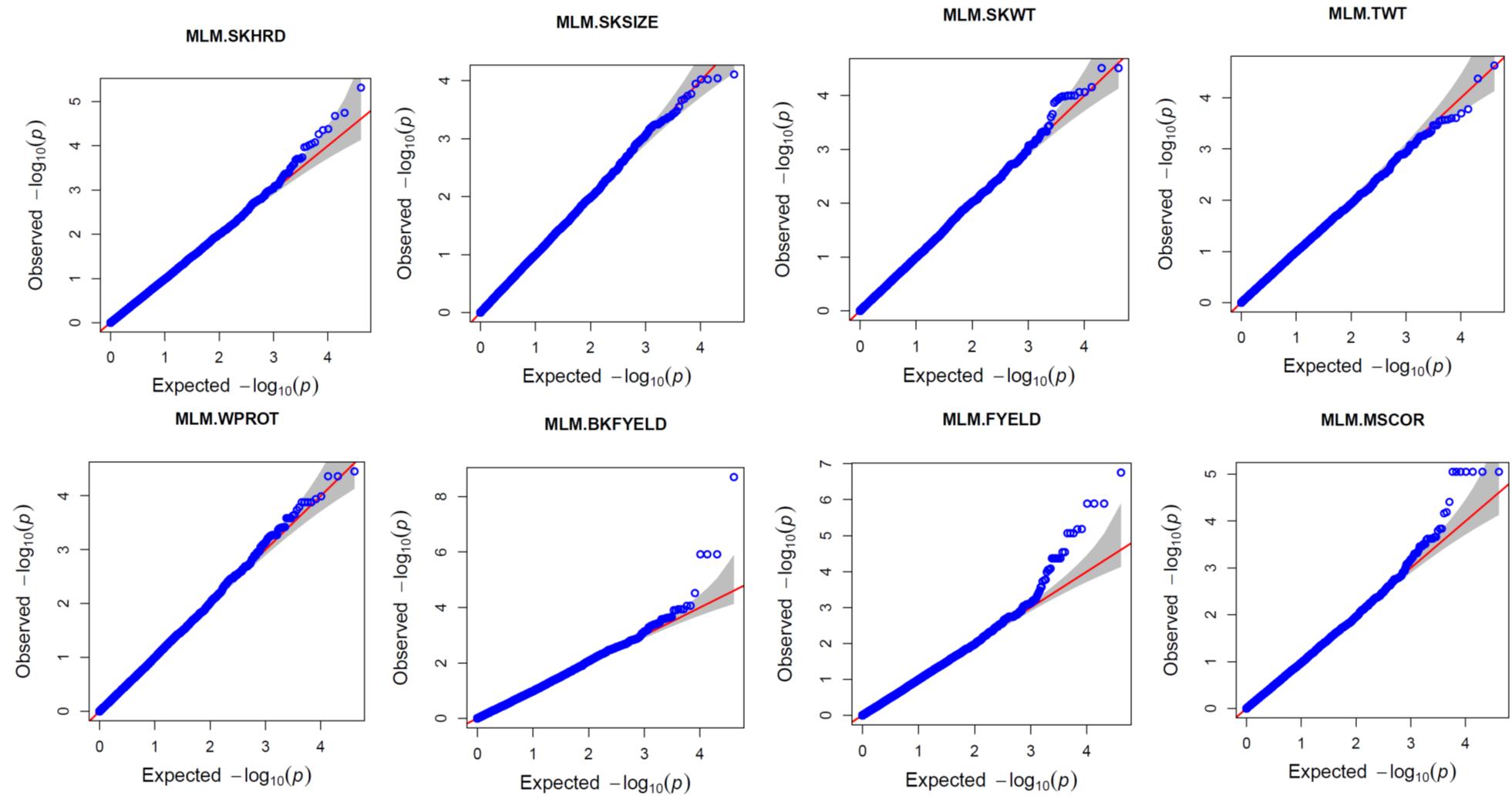


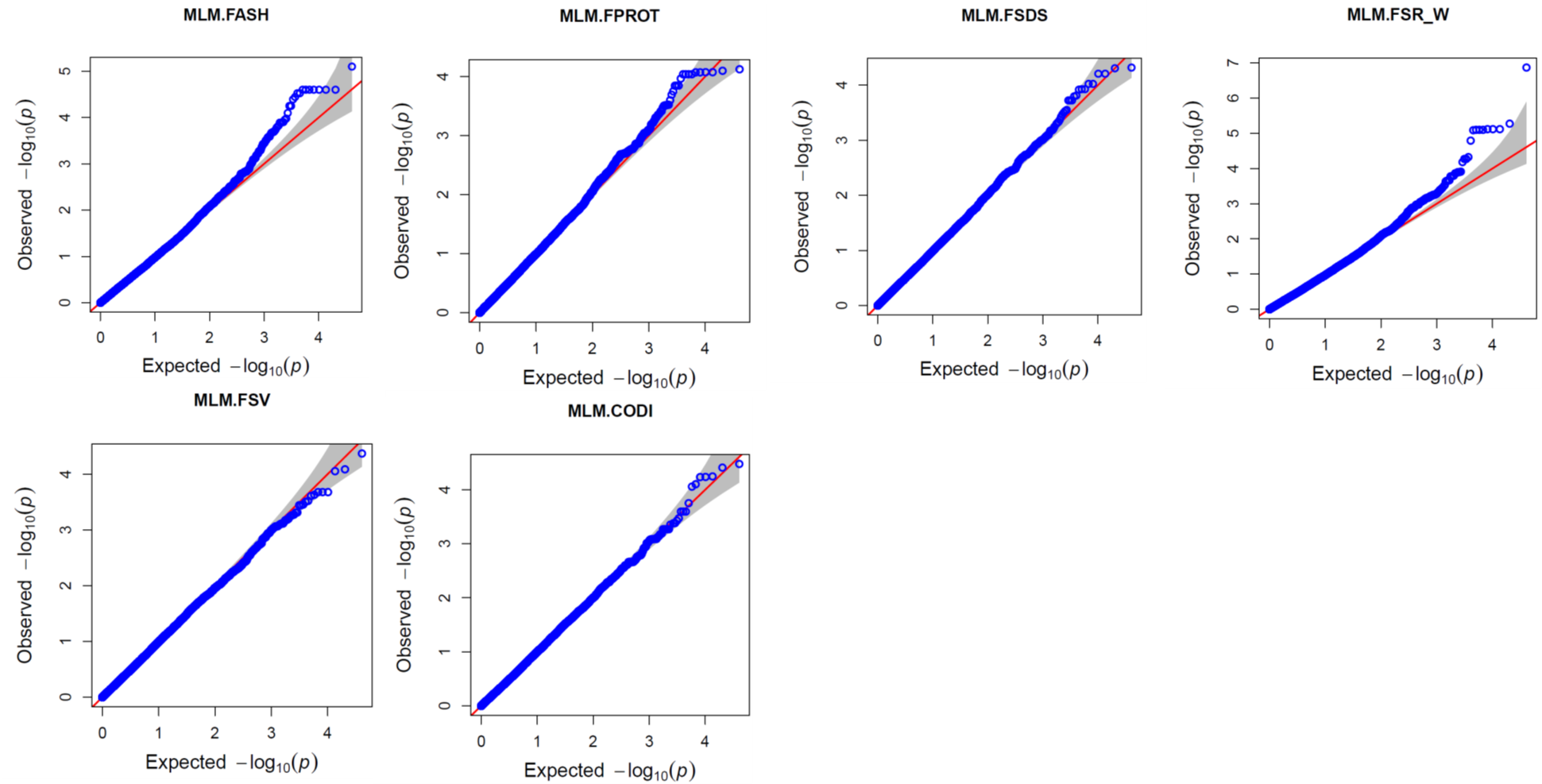


**Supplementary Fig. S4.** Quantile-Quantile plots of the expected -log_10_ (*P*) versus the observed -log_10_ (*P*) for association mapping model MLM for the 14 end-use quality traits.


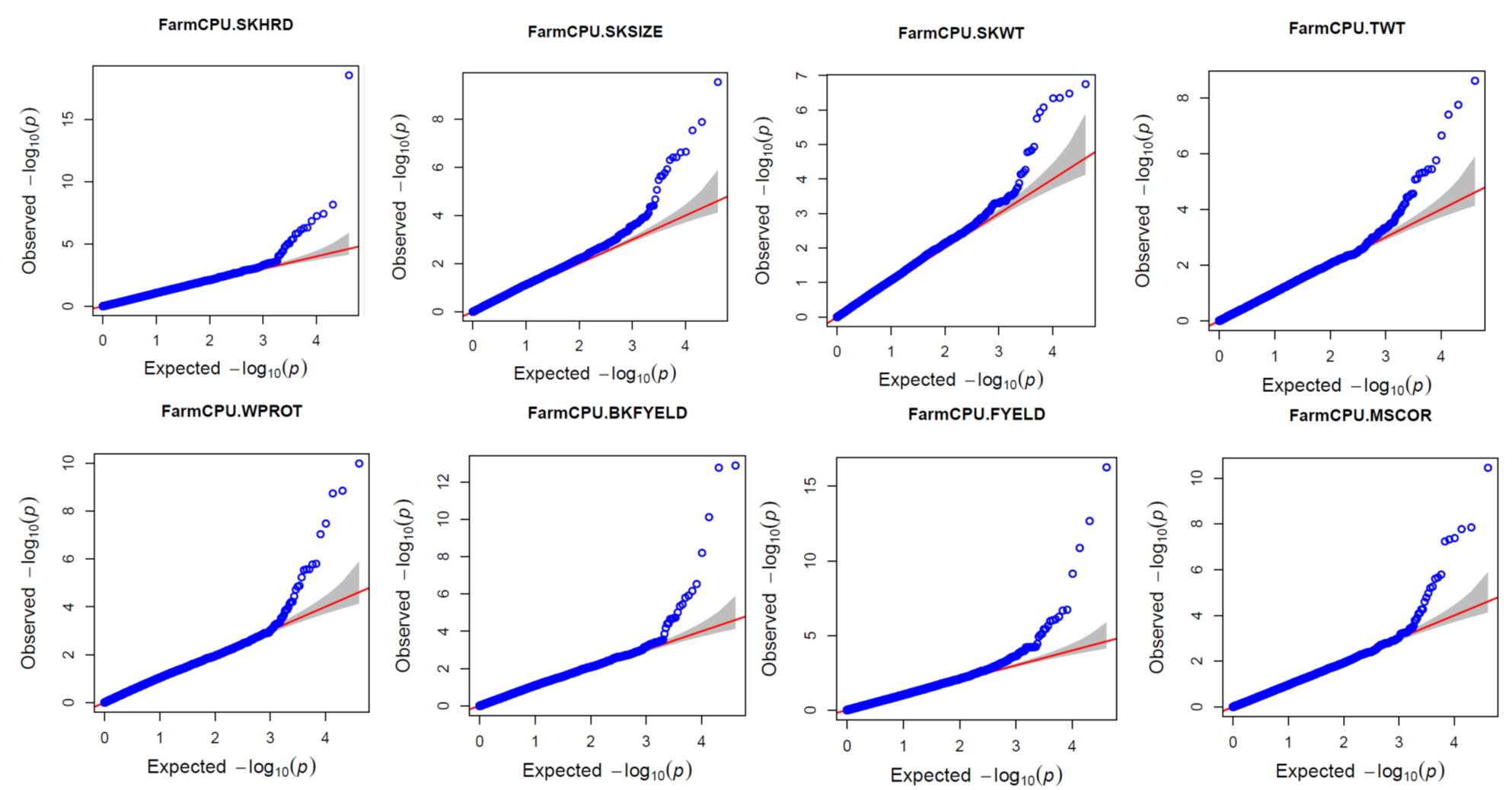


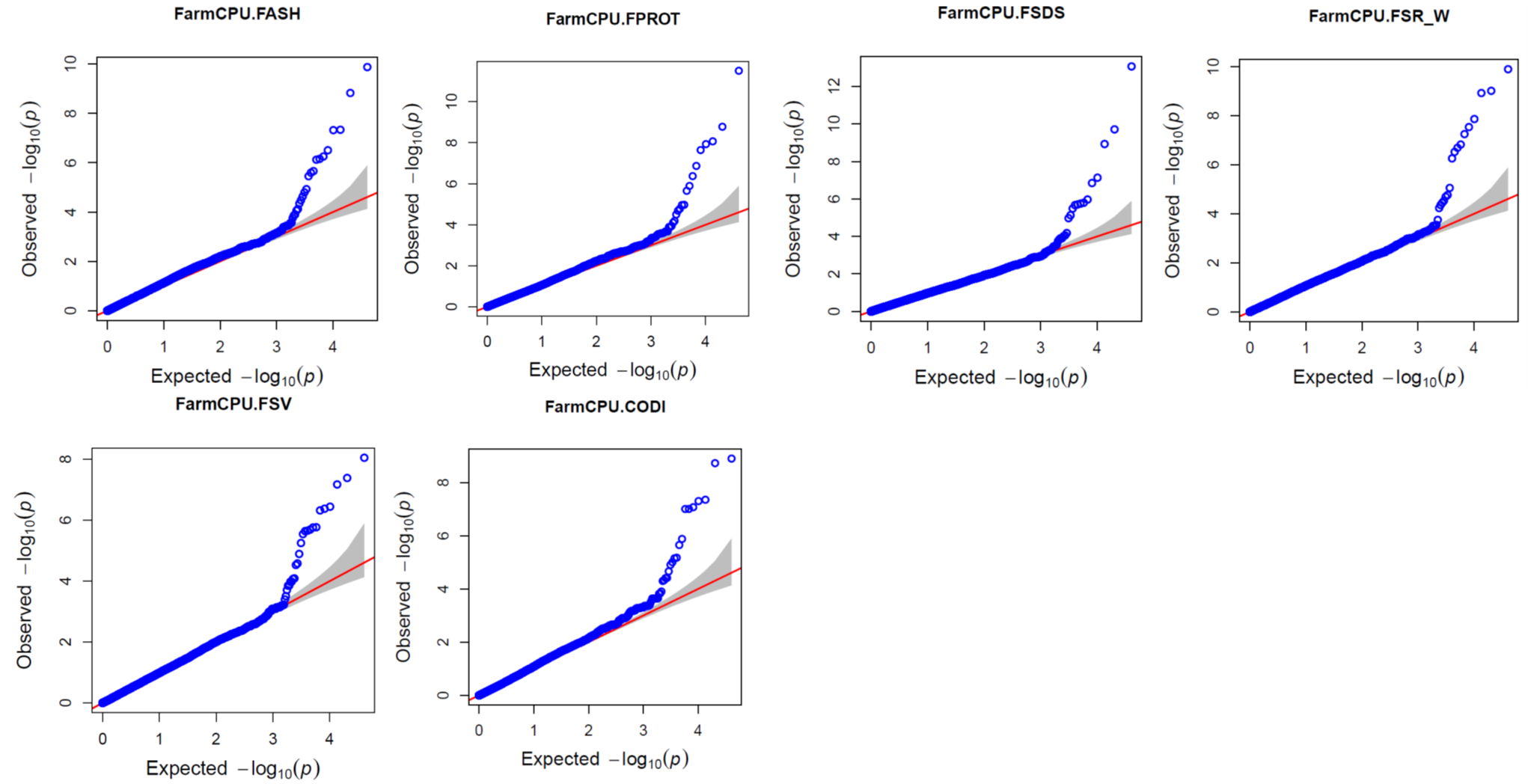


**Supplementary Fig. S5.** Quantile-Quantile plots of the expected -log_10_ (*P*) versus the observed -log_10_ (*P*) for association mapping model FarmCPU for the 14 end-use quality traits


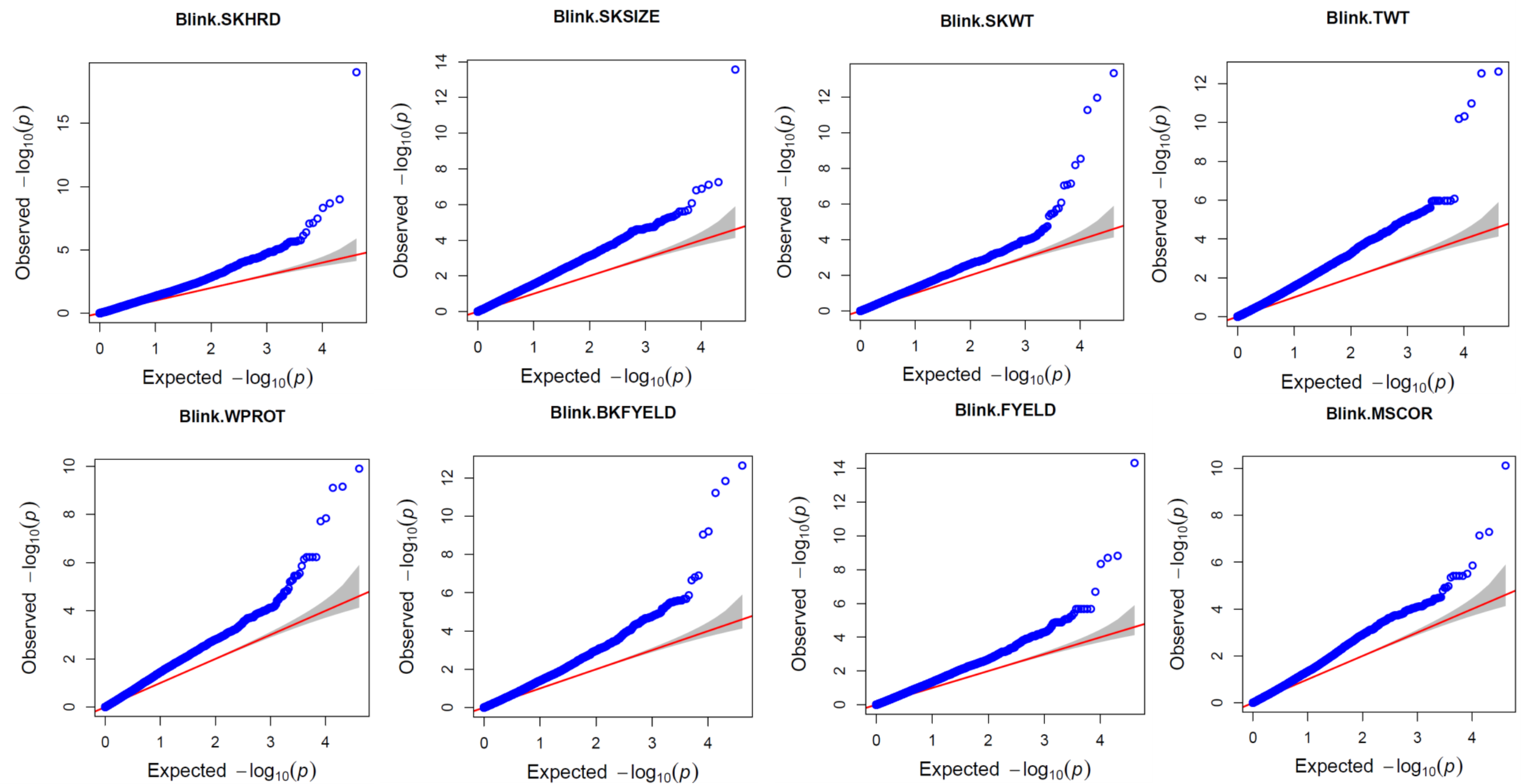


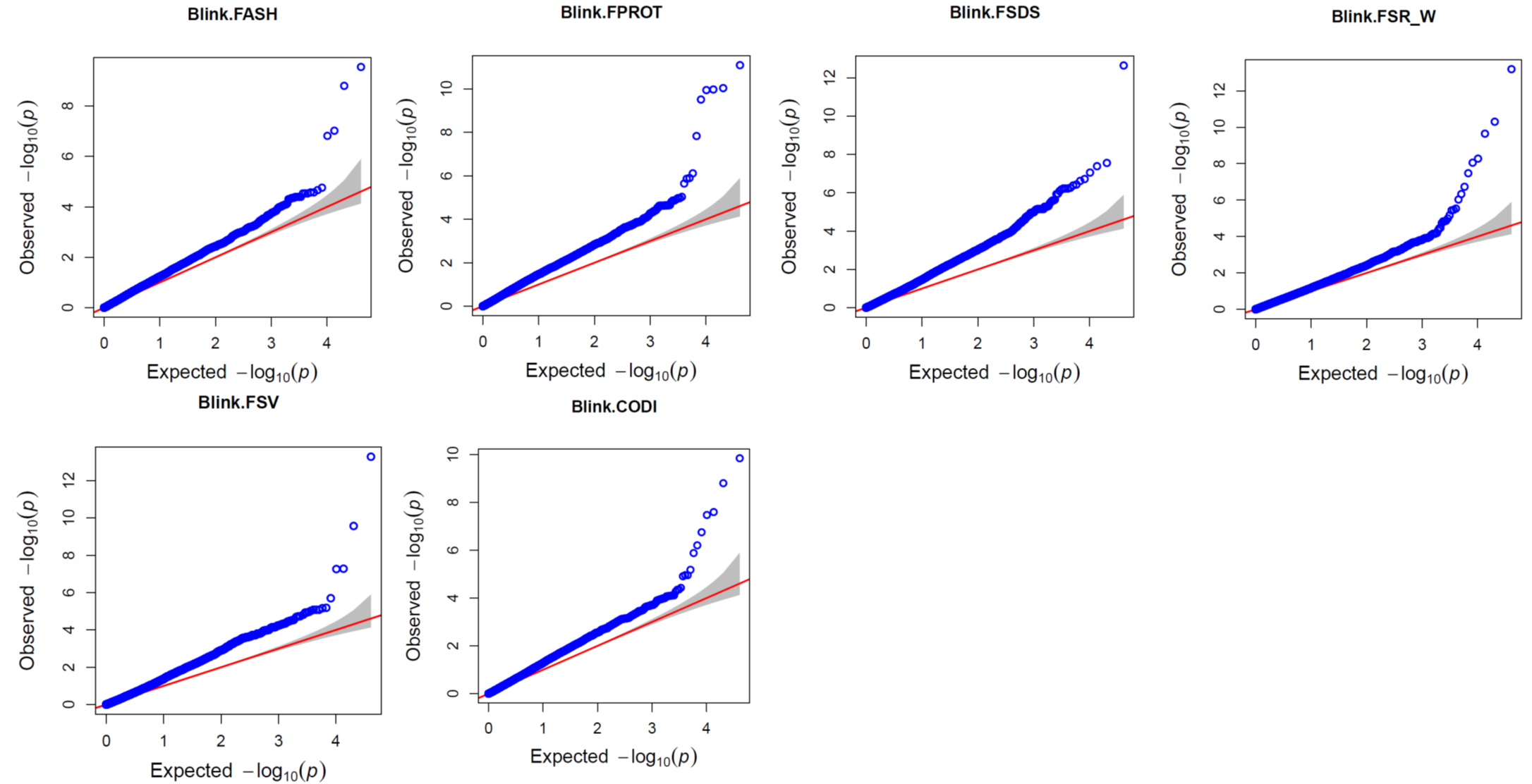


**Supplementary Fig. S6.** Quantile-Quantile plots of the expected -log_10_ (*P*) versus the observed -log_10_ (*P*) for association mapping model BLINK for the 14 end-use quality traits.


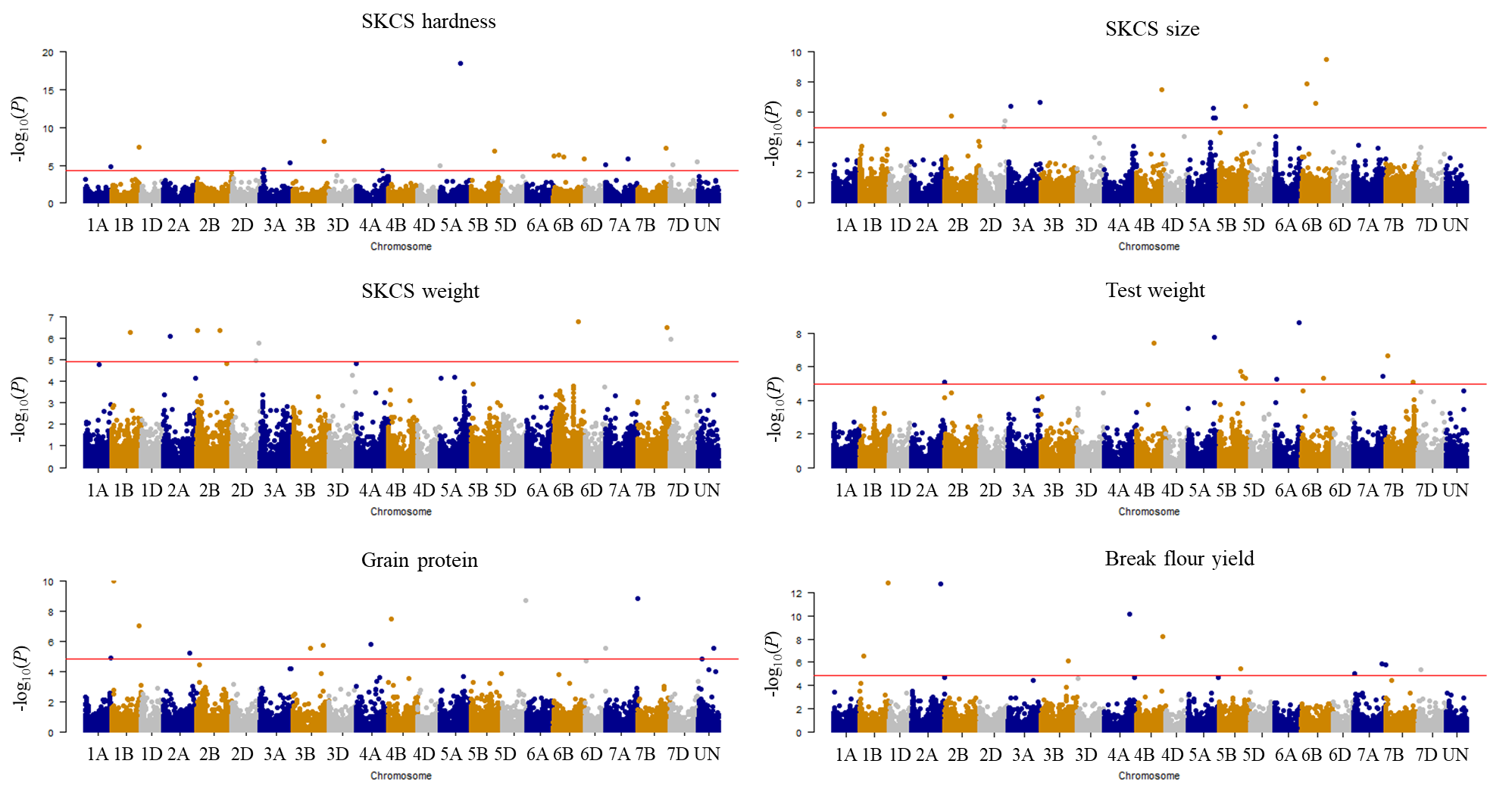


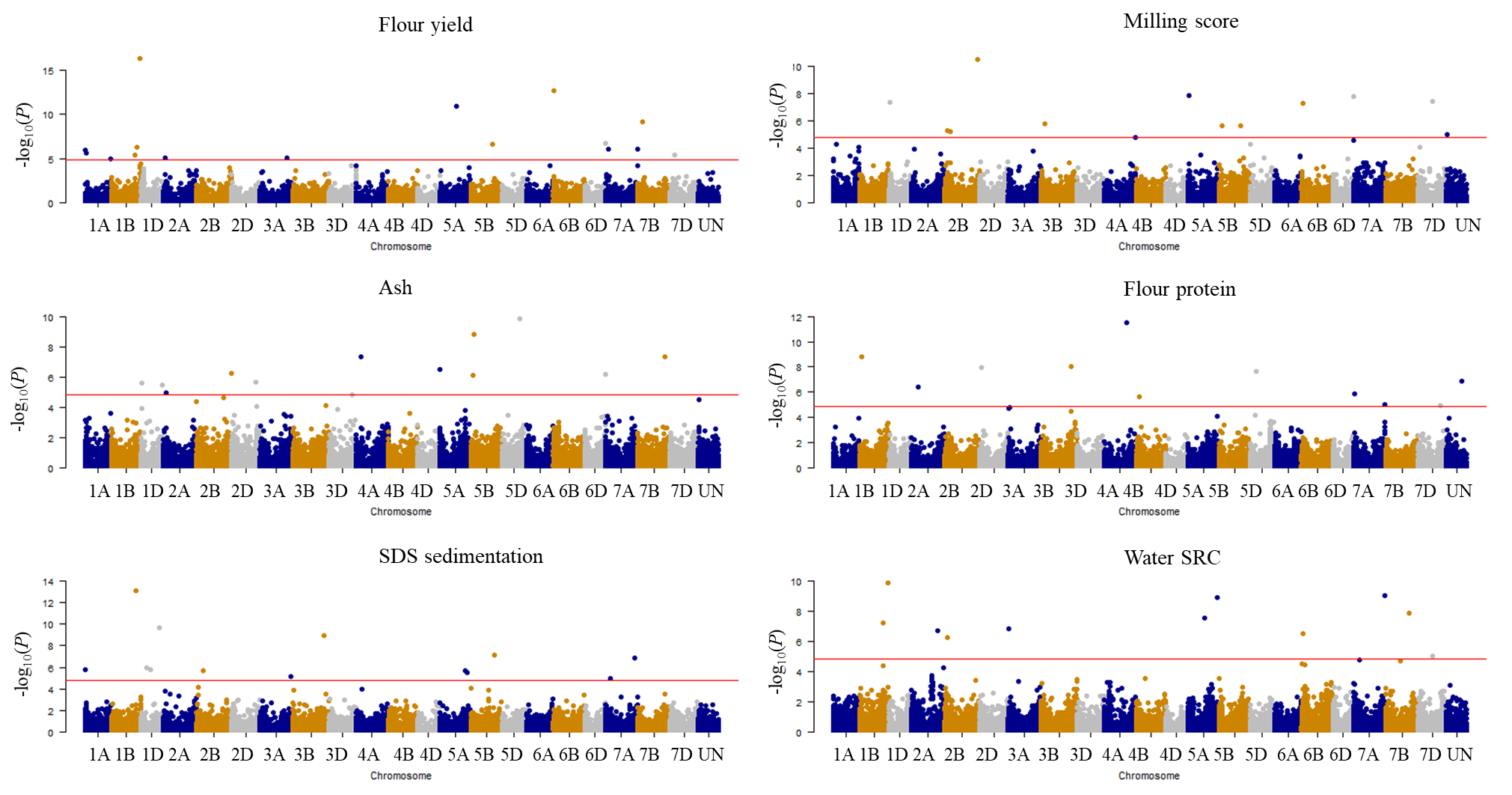


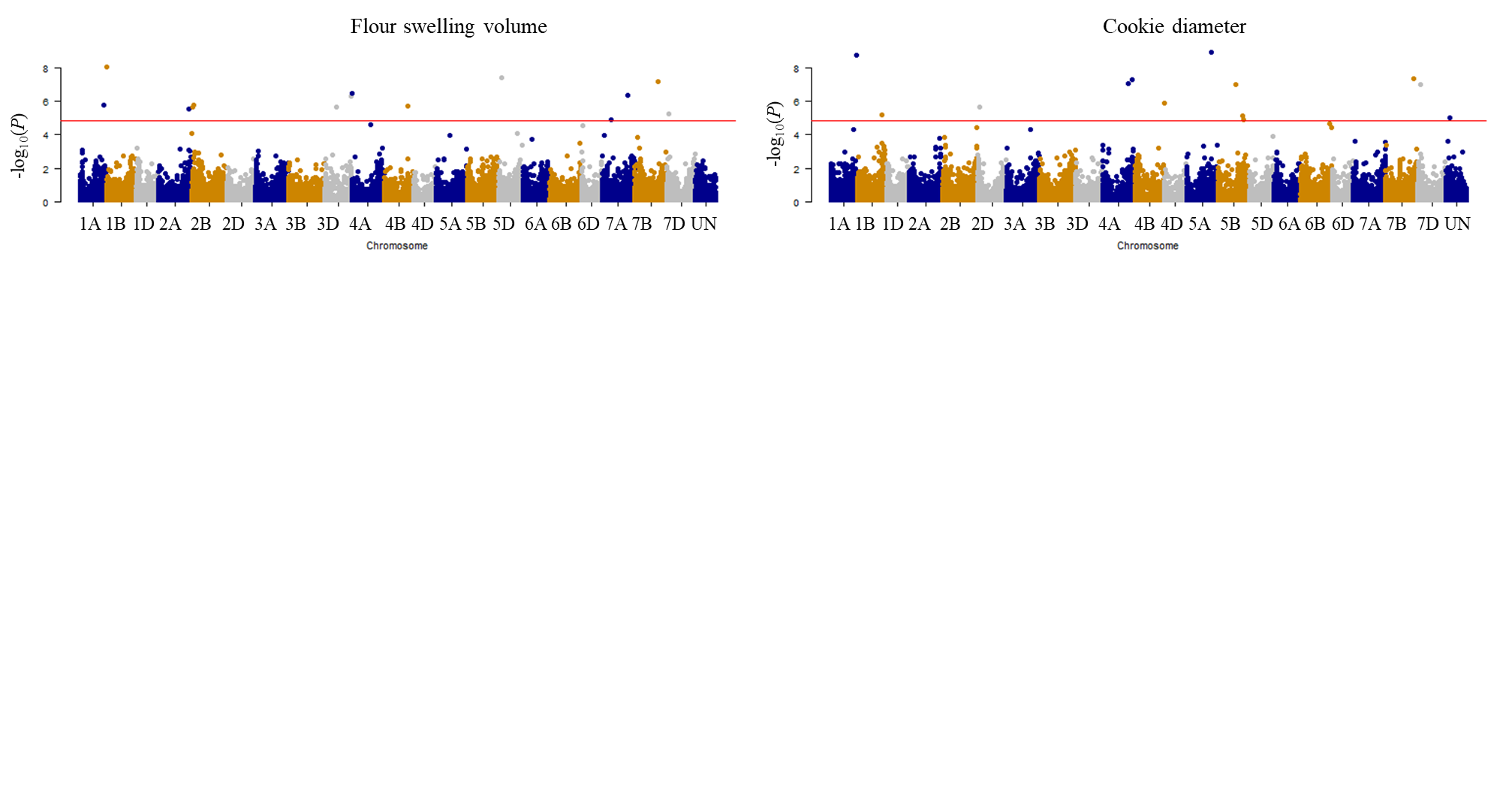
**Supplementary Fig. S7.** Summary of genome-wide association studies for 14 end-use quality traits in 672 soft winter wheat genotypes based on Fixed and random model Circulating Probability Unification model. The horizontal red line indicates significance level at FDR ≤ 0.05.
